# A Programmed Cell Death Protein 1 Pathway-Derived Prognostic Transcriptomic Signature in Bladder Cancer: Development in TCGA-BLCA and Evaluation in Independent Cohorts

**DOI:** 10.3390/cimb48101056

**Published:** 2026-10-13

**Authors:** Hao-Han Chang, Pai-Chi Teng, Chia-Hsin Liu, Yung-Lun Lin, Shu-Chi Wang, Bo-Ying Bao, Hsin-Chih Yeh, Chih-Pin Chuu, Jiun-Hung Geng, Li-Hsin Chang, Tsz-Yi Tang, Hsien-Che Ou, Shu-Pin Huang, Wei-Chung Cheng, Chia-Yang Li

**Affiliations:** 1 Graduate Institute of Clinical Medicine, College of Medicine, Kaohsiung Medical University, Kaohsiung 80708, Taiwan; ocean6316@gmail.com (H.-H.C.); martin@kmu.edu.tw (C.-H.L.); u9001090@hotmail.com (J.-H.G.);; 2 Department of Urology, Kaohsiung Medical University Hospital, Kaohsiung 80756, Taiwan; 3 Division of Urology, Department of Surgery, Cardinal Tien Hospital, New Taipei City 23148, Taiwan; paichi.teng@gmail.com; 4 Department of Urology, National Taiwan University Hospital, Taipei 10002, Taiwan; 5 Research Center for Cancer Biology, China Medical University, Taichung 40403, Taiwanwccheng@mail.cmu.edu.tw (W.-C.C.); 6 Department of Medical Laboratory Science and Biotechnology, Kaohsiung Medical University, Kaohsiung 80708, Taiwan; shuchiwang@kmu.edu.tw; 7 Department of Pharmacy, China Medical University, Taichung 40403, Taiwan; bao@mail.cmu.edu.tw; 8 Department of Nursing, Asia University, Taichung 41354, Taiwan; 9 Graduate Institute of Medicine, College of Medicine, Kaohsiung Medical University, Kaohsiung 80708, Taiwan; patrick1201.tw@yahoo.com.tw; 10 Department of Urology, School of Medicine, College of Medicine, Kaohsiung Medical University, Kaohsiung 80708, Taiwan; 11 Institute of Cellular and System Medicine, National Health Research Institutes, Miaoli 350401, Taiwan; cpchuu@nhri.org.tw; 12 Research Center for Environmental Medicine, Kaohsiung Medical University, Kaohsiung 80708, Taiwan; 13 Department of Urology, Kaohsiung Municipal Siaogang Hospital, Kaohsiung 81267, Taiwan; 14 Graduate Institute of Animal Vaccine Technology, National Pingtung University of Science and Technology, Pingtung 91201, Taiwan; cc1296@gmail.com; 15 Institute of Medical Science and Technology, College of Medicine, National Sun Yat-Sen University, Kaohsiung 804201, Taiwan; 16 Ph.D. Program for Cancer Biology and Drug Discovery, China Medical University and Academia Sinica, Taichung 40403, Taiwan; 17 Center of Disease Multi-Omics Research, Kaohsiung Medical University, Kaohsiung 80708, Taiwan; 18 Department of Medical Research, Kaohsiung Medical University Hospital, Kaohsiung 80756, Taiwan

**Keywords:** bladder cancer, programmed cell death protein 1, prognostic signature, external evaluation, immune checkpoint inhibitors, tumor microenvironment

## Abstract

Immune checkpoint inhibitors (ICIs) targeting programmed cell death protein 1 (PD-1) and programmed death-ligand 1 (PD-L1) have changed the treatment of advanced bladder cancer, but only a minority of patients benefit. We asked whether expression of PD-1 pathway genes carries prognostic information in bladder cancer. Sixty-eight genes annotated to the PD-1 signaling, CD28 co-stimulation, and cytotoxic T-lymphocyte-associated protein 4 (CTLA-4) inhibitory signaling pathways in Reactome were examined in The Cancer Genome Atlas bladder urothelial carcinoma cohort (TCGA-BLCA). Genes differentially expressed between tumor and normal tissue were entered into a least absolute shrinkage and selection operator (LASSO) Cox model, yielding a seven-gene risk score (*FYN*, *GRAP2*, *TRIB3*, *MAP3K8*, *AKT3*, *CD274*, and *CD80*). In TCGA-BLCA (*n* = 404), the risk score was associated with overall survival independently of age, sex, and stage (hazard ratio [HR] per standard deviation 1.24, 95% confidence interval [CI] 1.11–1.39) and modestly improved discrimination over clinical variables (concordance index [C-index] 0.630 to 0.664). With coefficients and standardization fixed in advance, the score was not associated with cancer-specific survival in GSE13507 (*n* = 165; HR per standard deviation 1.21, 95% CI 0.83–1.77) or with outcome in four further independent cohorts, including an atezolizumab-treated cohort; in a second, smaller ICI-treated cohort it was associated with shorter survival. Gene selection was unstable under bootstrap resampling, and the signature’s associations with transcriptome-inferred immune indices were limited. This PD-1 pathway-derived signature should therefore be regarded as exploratory; its prognostic value outside TCGA-BLCA is not supported by the available evidence.

## 1. Introduction

Bladder cancer is the ninth most commonly diagnosed malignancy worldwide, with an estimated 613,791 new cases and 220,349 deaths annually [1]. Established risk factors include tobacco smoking and occupational exposure to paint components, diesel exhaust, combustion products in firefighting, and hairdressing chemicals [2,3,4]. However, many patients with bladder cancer, which in this article refers to urothelial carcinoma of the bladder, have no apparent risk factors [5]. Platinum-based chemotherapy has long been the mainstay of systemic treatment in muscle-invasive and advanced bladder cancer [6]. Nevertheless, outcomes are poor, particularly in metastatic disease, with a median overall survival (OS) of less than two years [7,8].

Over the past decade, immune checkpoint inhibitors (ICIs) targeting programmed cell death protein 1 (PD-1, e.g., pembrolizumab [9,10] and nivolumab [11]), programmed death-ligand 1 (PD-L1, e.g., atezolizumab [12,13], avelumab [14,15], and durvalumab [16]), and cytotoxic T-lymphocyte-associated protein 4 (CTLA-4, e.g., ipilimumab [17]) have changed the treatment of bladder cancer. However, only a subset of patients benefits from ICIs, and some patients experience early progression or death. For example, early deaths were observed in the IMvigor210 trial among patients with PD-L1 expression below 5% [12], and in the KEYNOTE-052 trial among patients with a PD-L1 combined positive score (based on PD-L1-positive tumor and infiltrating immune cells) below 10 [9]. In addition, several phase 3 trials of first-line ICIs in advanced bladder cancer did not show the expected benefit [18,19].

ICIs are used as maintenance therapy after platinum-based chemotherapy, as later-line therapy, and in platinum-ineligible patients [6], and more recently in combination with enfortumab vedotin as first-line treatment [20]. Nevertheless, only a minority of patients derive durable benefit, and biomarkers that reflect PD-1 pathway biology remain an unmet need. The most commonly used biomarker is based on tumor PD-L1 expression [21]. PD-L1 expression can be measured by immunohistochemistry, Western blotting, polymerase chain reaction (PCR), or microarray, and the results vary considerably across methods [21,22]. Other candidate markers include microsatellite instability (MSI) [23], tumor mutational burden [24], TGF-β [25], and CD8+ T-cell infiltration [26], but none has yet entered routine clinical practice. Biomarkers that summarize PD-1 pathway biology more broadly than a single protein may therefore be informative.

In this study, we aimed to identify PD-1 pathway genes associated with survival in bladder cancer using public transcriptomic databases. Using differential expression analysis followed by least absolute shrinkage and selection operator (LASSO) Cox regression, we developed a seven-gene risk score consisting of *FYN*, *GRAP2*, *TRIB3*, *MAP3K8*, *AKT3*, *CD274*, and *CD80* in The Cancer Genome Atlas bladder urothelial carcinoma cohort (TCGA-BLCA), examined its discrimination, calibration, and stability, and evaluated it with a prespecified procedure in independent cohorts. We also examined its associations with transcriptome-inferred indices of the tumor immune microenvironment that have been associated with responses to ICIs [27,28,29].

## 2. Materials and Methods

### 2.1. Data Collection and Preparation

RNA sequencing (RNA-seq) profiles were obtained from the DriverDB database [30,31,32,33]. The mRNA expression data of primary tumors and normal tissues in DriverDB were retrieved from the TCGA data portal in 2018 [32,33], and genes were annotated with Ensembl release 101 [32,33]. Expression values of 20,495 genes, as upper-quartile-normalized fragments per kilobase of transcript per million mapped reads (FPKM-UQ), were used for signature construction. Clinical data were obtained from the TCGA Clinical Data Resource (TCGA-CDR) [34]. Clinical data and RNA-seq profiles were matched by TCGA barcode (https://docs.gdc.cancer.gov/Encyclopedia/pages/TCGA_Barcode/, accessed on 12 October 2026). In total, 406 bladder cancer (BLCA) tumors and 19 normal tissues were retrieved for further analysis. The seven-gene model was derived from these data. Because the intermediate files of the original analysis were no longer available at the time of peer review, all results reported in this version were re-derived from publicly available data using the code provided in the Supplementary Materials: TCGA-BLCA expression data were obtained from the Genomic Data Commons (STAR FPKM-UQ), and 404 primary tumors had both expression and overall survival data.

### 2.2. PD-1 Pathway-Relevant Gene Selection

Pathways whose names contained the keywords “CD28 co-stimulation”, “PD-1 signaling”, or “CTLA4 inhibitory signaling” were identified in the Reactome database [35]. Three pathways matched: (1) CD28 co-stimulation, (2) CTLA4 inhibitory signaling, and (3) PD-1 signaling. The 68 genes belonging to these pathways were selected.

### 2.3. Differential Expression

Differential expression between primary tumors and normal bladder tissue was assessed with DESeq2 (version 1.52.0) [36] using raw STAR-aligned read counts obtained from the Genomic Data Commons (404 primary tumors and 19 normal tissues with available count data), as DESeq2 requires unnormalized counts. Genes with |log_2_ fold change| > 1 and a Benjamini–Hochberg-adjusted *p*-value (false discovery rate, q-value) < 0.05 were defined as differentially expressed genes (DEGs). Restricting the candidates to genes differentially expressed between tumors and normal tissue was intended to limit the number of candidate genes relative to the number of events and to favor genes whose expression is altered in bladder cancer; prognostic genes without tumor-normal differences may therefore have been excluded.

### 2.4. Feature Selection and Signature Construction

The least absolute shrinkage and selection operator (LASSO) was used to select genes for the signature [37,38]. The R package glmnet was used to fit a LASSO-penalized Cox regression of overall survival on the DEGs, with the penalty parameter λ selected by 10-fold cross-validation as the value minimizing the partial-likelihood deviance (lambda.min). Expression values were standardized before model fitting, and the risk score was computed on the same scale.

The risk score of each patient was calculated as the sum of the standardized expression values of the selected genes weighted by their LASSO coefficients

(1) $$\mathrm{Risk}\;\mathrm{score}=\sum_{i=1}^{n}Coef(i)*x(i)$$

where Coef(*i*) is the LASSO coefficient of gene *i* (Table 1), x(*i*) is the expression of gene *i* standardized to zero mean and unit variance within the cohort, and *n* = 7 is the number of genes in the signature.

For descriptive analyses, patients were divided into high- and low-risk groups at the median risk score within each cohort; in external cohorts, the training-cohort median threshold on the standardized scale was also applied unchanged. The risk score was otherwise analyzed as a continuous variable.

Model performance was assessed in TCGA-BLCA using Harrell’s concordance index (C-index) and time-dependent areas under the receiver operating characteristic curve (AUC) at 1, 3, and 5 years. The incremental value of the risk score was assessed by comparing a clinical Cox model (age ≥ 65 years, sex, and stage III/IV vs. I/II) with the same model plus the risk score, using the likelihood-ratio test and the difference in C-index with a bootstrap 95% confidence interval (1000 resamples). The optimism-corrected C-index and calibration slope of the combined model, and its calibration for 3-year overall survival, were estimated with 500 bootstrap resamples. The risk score was also analyzed as a continuous variable (hazard ratio per standard deviation); its functional form was examined with a restricted cubic spline with four knots, and the proportional-hazards assumption was checked with Schoenfeld residuals. To assess the stability of gene selection, the complete modeling pipeline (10-fold cross-validated LASSO-Cox regression with lambda.min applied to the nine candidate genes) was repeated in 500 bootstrap resamples, recording how often each gene was selected and estimating the optimism-corrected C-index of the pipeline. For these analyses, expression data were re-derived from the Genomic Data Commons (STAR FPKM-UQ; 404 primary tumors with overall survival data, of which 402 had complete stage information), and the published coefficients were applied unchanged. These analyses were performed in R 4.6.0 with the packages survival 3.8–6, glmnet 5.0, rms 8.1–1, timeROC 0.4.1, pROC 1.19.0.1, and survminer 0.5.2, using random seed 2026.

### 2.5. Independent External Evaluation

The prespecified external cohort was GSE13507 from the Gene Expression Omnibus (165 primary tumors with expression and clinical data; Illumina HumanRef-8 v2, one probe for each of the seven genes) [39]. Cancer-specific survival was the primary endpoint in this cohort because it was the endpoint reported by the original investigators; overall survival was a secondary endpoint. To address the generalizability of the signature, it was additionally evaluated in all further publicly available bladder cancer cohorts with expression and survival data that we had processed: GSE31684 (93 muscle-invasive tumors treated by cystectomy; overall survival), GSE48075 (73 muscle-invasive tumors; overall survival), GSE32894 (224 mainly non-muscle-invasive tumors; disease-specific survival), and two cohorts of metastatic urothelial carcinoma treated with PD-1/PD-L1 inhibitors, IMvigor210 (348 patients; overall survival and objective response) and GSE176307 (88 patients; overall survival and objective response). The procedure was fixed before evaluation: the published coefficients were not re-estimated; expression values on each platform’s linear scale (FPKM-UQ or upper-quartile-normalized counts for RNA sequencing, and 2 raised to the log_2_ intensity for microarrays) were standardized per gene within each cohort and combined with the coefficients. The prespecified primary analysis was the hazard ratio per standard deviation of the risk score; log_2_-scale expression was used as a sensitivity analysis for GSE13507. Discrimination was assessed with Harrell’s C-index and, for objective response in the ICI-treated cohorts, with the area under the receiver operating characteristic curve, with the direction prespecified (higher risk score, lower response).

### 2.6. Evaluation of Stromal Content, Immune Cell Infiltration, and Tumor Purity

ESTIMATE (Estimation of STromal and Immune cells in MAlignant Tumor tissues using Expression data), which infers stromal and immune cell content and tumor purity from expression data, was used to characterize the tumor microenvironment in TCGA-BLCA [28]. Immune scores, stromal scores, ESTIMATE scores, and tumor purity were compared between the high-risk and low-risk groups. Groups were compared with the Wilcoxon rank-sum test, and the *p*-values of the six indices (four ESTIMATE indices in TCGA-BLCA and two TIDE indices in GSE13507) were adjusted with the Benjamini–Hochberg procedure (q < 0.05 considered significant). ESTIMATE scores were computed with the R package estimate (version 1.0.13).

### 2.7. Tumor Immune Dysfunction and Exclusion Analysis

Tumor Immune Dysfunction and Exclusion (TIDE), a computational framework that models T-cell dysfunction and exclusion as surrogates of tumor immune evasion, was applied to GSE13507 [29]. The TIDE score and the TIDE-predicted microsatellite instability (MSI) score were compared between the high-risk and low-risk groups as described in Section 2.6. TIDE scores were computed with the TIDE command-line implementation (tidepy 1.3.9; cancer type “Other”) from log_2_ expression values centered on the mean of each gene across samples.

### 2.8. Comparison of Immune Cell Proportions

The original analysis used CIBERSORT [40] to estimate immune-cell proportions. Because the licensed CIBERSORT source code and LM22 signature matrix could not be obtained for the re-analysis, and so that all analyses can be reproduced without licensed software, immune-cell composition in TCGA-BLCA was estimated with two open-source methods: quanTIseq [41], which estimates the fractions of ten immune-cell types from linear transcripts per million (TPM) values (TIL10 signature), and MCP-counter [42], which estimates the abundance of eight immune and two stromal cell populations from log_2_(TPM + 1) values. Values were compared between risk groups with the Wilcoxon rank-sum test, and *p*-values were adjusted with the Benjamini–Hochberg procedure within each method. quanTIseq and MCP-counter were run with the R packages quantiseqr (version 1.20.0) and MCPcounter (version 1.2.0).

### 2.9. Use of Generative Artificial Intelligence

During the preparation of this manuscript and its revision, the authors used Claude (Anthropic; Claude Fable 5.1 and Claude Opus 5.5) to assist with editing the text and with writing and running the R and Python code for the re-analysis. All code and outputs were reviewed by the authors, and the complete code needed to reproduce every result in this version, together with software versions and random seeds, is provided in the Supplementary Materials. The authors take full responsibility for the content of this publication.

## 3. Results

### 3.1. Identification of PD-1 Pathway-Associated Genes in Bladder Cancer

**Figure 1:**
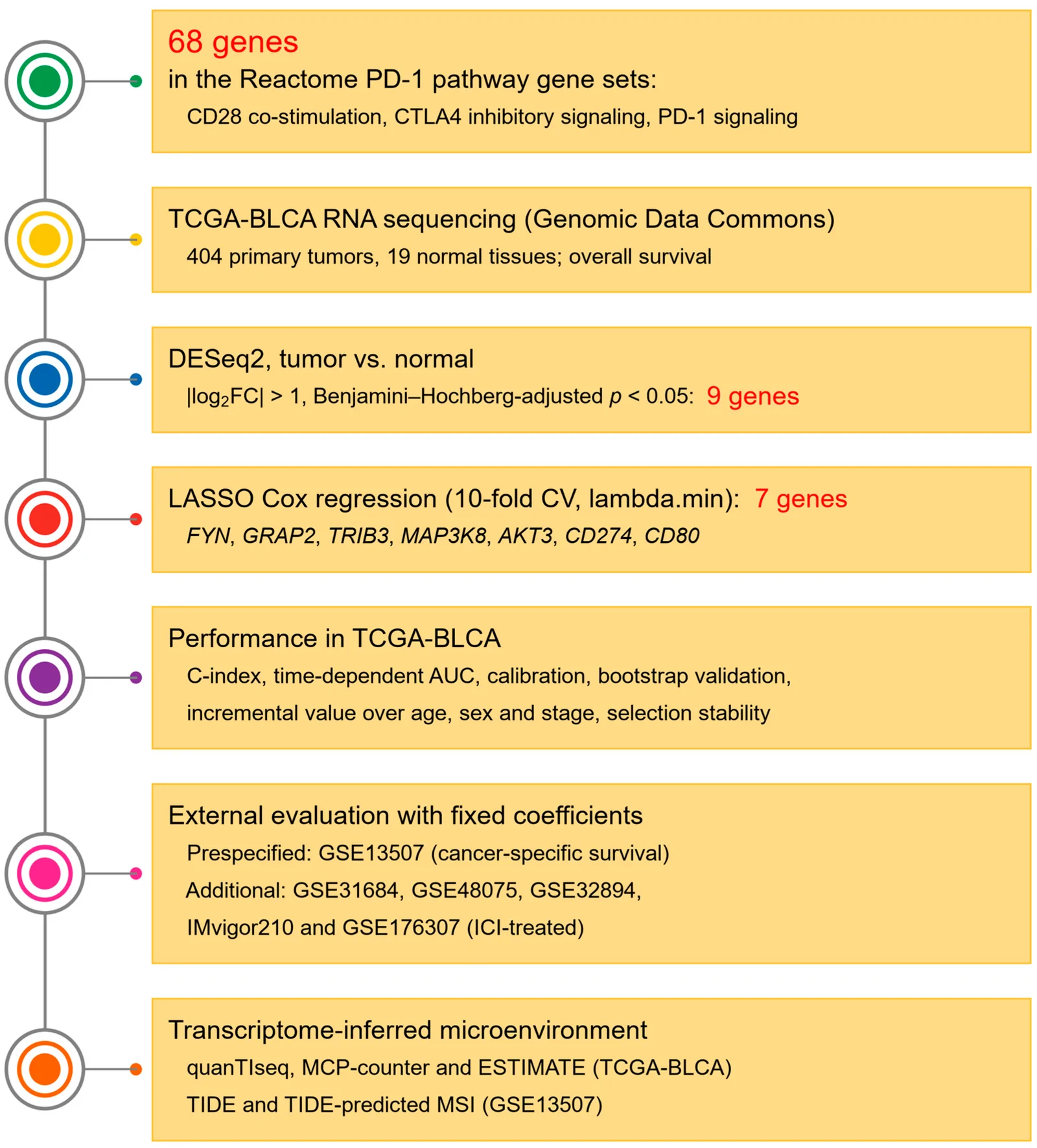
Workflow of the study: selection of PD-1 pathway genes, construction of the seven-gene risk score in TCGA-BLCA, and its evaluation in independent cohorts. Numbers in red indicate the number of genes retained at each step. Abbreviations: CV, cross-validation; FC, fold change; ICI, immune checkpoint inhibitor; MSI, microsatellite instability; TIDE, Tumor Immune Dysfunction and Exclusion.

Three Reactome pathways related to PD-1 biology were identified [35], including CD28 co-stimulation, CTLA-4 inhibitory signaling, and PD-1 signaling (Figure 1). Together, these pathways contained 68 genes. Gene expression and clinical data were obtained from The Cancer Genome Atlas Urothelial Bladder Carcinoma (TCGA-BLCA) cohort [43]. Expression of each of the 68 genes was compared between tumors and normal bladder tissue (Table S1). Nine genes were differentially expressed (|log_2_ fold change| > 1 and Benjamini–Hochberg-adjusted *p* < 0.05): *FYN*, *GRAP2*, *TRIB3*, *MAP3K8*, *AKT3*, *CD274*, *CD80*, *PIK3R1*, and *CD28*. These DEGs were annotated using the Reactome [35] and the Kyoto Encyclopedia of Genes and Genomes (KEGG) [44] databases (Figures S1 and S2). In Reactome, eight of the nine genes mapped to the CTLA-4 inhibitory signaling and CD28 co-stimulation pathways. KEGG analysis showed enrichment of the PD-1 signaling pathway (*CD274*) and the PD-L1 expression and PD-1 checkpoint pathway in cancer (*CD274*, *CD28*, *PIK3R1*, and *AKT3*).

### 3.2. Construction of the Seven-Gene Risk Score for Overall Survival

**Figure 2:**
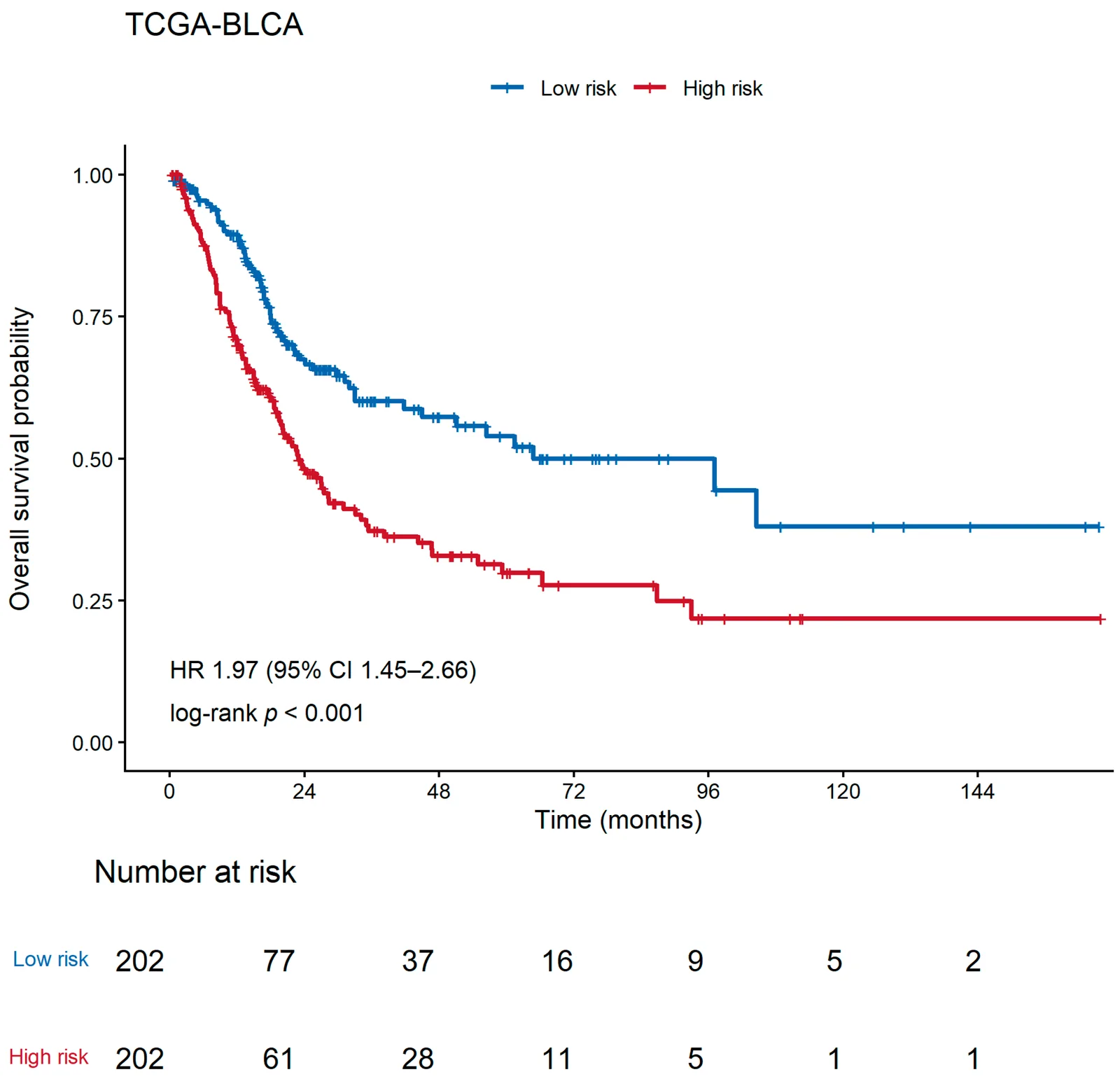
Kaplan–Meier analysis of overall survival by risk group in the training cohort (TCGA-BLCA, *n* = 404), re-derived from Genomic Data Commons data. Patients were divided at the median risk score. The hazard ratio (HR) with 95% confidence interval (CI) from a univariable Cox model and the log-rank *p*-value are shown; the table gives the number of patients at risk. Time is shown in months.

In TCGA-BLCA, 406 patients had OS data and expression data for the nine DEGs. LASSO Cox regression was used for feature selection and coefficient estimation. Seven genes were selected (*FYN*, *GRAP2*, *TRIB3*, *MAP3K8*, *AKT3*, *CD274*, and *CD80*); their log_2_ fold changes and LASSO coefficients are shown in Table 1. Genes with positive coefficients increase the risk score, which corresponds to shorter OS. Patients were divided into high-risk and low-risk groups at the median risk score. Kaplan–Meier analysis showed that patients with higher risk scores had shorter OS (Figure 2; in the re-derived dataset of 404 patients, hazard ratio (HR) = 1.97, 95% confidence interval (CI) 1.45 to 2.66, log-rank *p* < 0.001).

### 3.3. External Evaluation of the Seven-Gene Risk Score in an Independent Cohort

**Figure 3:**
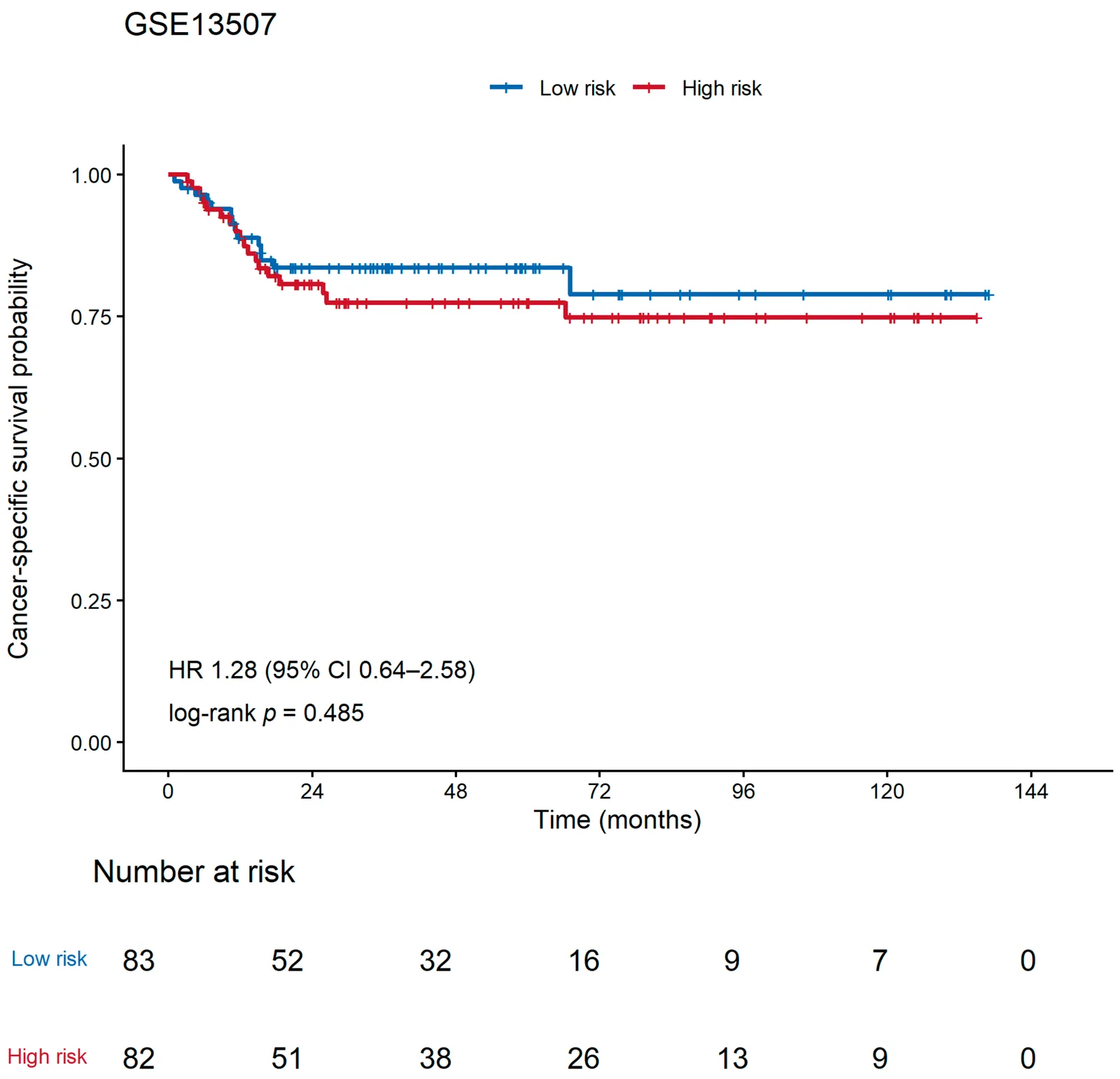
Kaplan–Meier analysis of cancer-specific survival by risk group in the external evaluation cohort (GSE13507, *n* = 165; 32 cancer-specific deaths). Patients were divided at the median risk score. The HR with 95% CI from a univariable Cox model and the log-rank *p*-value are shown; the table gives the number of patients at risk. Time is shown in months.

We then evaluated the seven-gene risk score in GSE13507, which included primary bladder cancer samples from 165 patients with clinical and transcriptomic information [39]. Patient characteristics by risk group are shown in Table 2. GSE13507 consisted predominantly of non-muscle-invasive (stage I) tumors, whereas TCGA-BLCA consisted mainly of muscle-invasive tumors. High-risk patients had a more advanced stage in both cohorts (TCGA-BLCA *p* = 0.003; GSE13507 *p* = 0.010) and were older in TCGA-BLCA (*p* = 0.021), whereas sex did not differ. In GSE13507, the risk score was not significantly associated with cancer-specific survival (32 deaths): the hazard ratio was 1.28 (95% CI 0.64 to 2.58; log-rank *p* = 0.485) for high versus low risk (Figure 3) and 1.21 (0.83 to 1.77; *p* = 0.318) per standard deviation. Applying the training-cohort threshold (HR 1.64, 0.79 to 3.41) or log_2_-scale expression (HR per standard deviation 1.27, 0.87 to 1.85) gave similar results, as did overall survival as the endpoint (HR per standard deviation 1.09, 0.83 to 1.43).

### 3.4. Cox Regression Analyses

Univariate and multivariate Cox regression analyses were conducted to adjust for possible confounding variables. In TCGA-BLCA (Table 3), high risk score, age ≥ 65 years, and stage III/IV were associated with worse OS, and the risk score remained independently associated with OS after adjustment for age, sex, and stage (HR 1.75, 95% CI 1.28 to 2.39, *p* < 0.001; 402 patients with complete data, 176 deaths). In GSE13507 (Table 4), age ≥ 65 years and stage II-IV were associated with shorter cancer-specific survival, whereas the risk score was not, in either univariate (HR 1.28, 0.64 to 2.58, *p* = 0.486) or multivariate analysis (HR 0.96, 0.47 to 1.96, *p* = 0.905). Stage was the dominant prognostic factor in this predominantly non-muscle-invasive cohort.

### 3.5. Discrimination, Calibration, and Stability of the Risk Score

To assess prognostic performance beyond association with survival, we re-derived the TCGA-BLCA expression data from the Genomic Data Commons and applied the published coefficients unchanged (404 patients and 177 deaths; 402 patients with complete stage information and 176 deaths). In this dataset, the dichotomized risk score was associated with overall survival after adjustment for age, sex, and stage (HR 1.75, 95% CI 1.28–2.39), consistent with the original analysis. As a continuous variable, each standard-deviation increase in the risk score was associated with an HR of 1.30 (95% CI 1.17–1.45) in univariable analysis and 1.24 (1.11–1.39) after adjustment (both *p* < 0.001), with some departure from linearity (*p* = 0.037; Figure S3) and no violation of proportional hazards (*p* = 0.713). The C-index was 0.618 (95% CI 0.575–0.662) for the risk score alone, 0.630 (0.590–0.670) for the clinical model, and 0.664 (0.624–0.705) for the clinical model plus the risk score (likelihood-ratio *p* < 0.001; difference in C-index 0.035, 95% CI 0.010–0.061; Table S2). Time-dependent AUCs of the combined model were 0.715, 0.668, and 0.683 at 1, 3, and 5 years, compared with 0.659, 0.630, and 0.634 for the clinical model alone. After bootstrap internal validation, the optimism-corrected C-index of the combined model was 0.658 and the calibration slope was 0.912; calibration for 3-year overall survival was satisfactory (Figure S4). In stage-stratified analyses, the HR per standard deviation was 1.25 (1.11–1.40) in stage III/IV and 1.17 (0.81–1.69) in stage I/II disease, with no evidence of interaction (*p* = 0.904); the wider interval in stage I/II disease reflects its smaller number of events (35). In 500 bootstrap resamples of the complete LASSO pipeline, *TRIB3* (91.2%), *MAP3K8* (82.2%), and *CD274* (78.0%) were selected most consistently, whereas *GRAP2* (52.2%) was selected about as often as *PIK3R1* (52.8%), which was not part of the final panel; the exact seven-gene set was reproduced in 3.0% of resamples, and the optimism-corrected C-index of the pipeline was 0.577 (Table S3). In GSE13507, the C-index of the risk score for cancer-specific survival was 0.583 (95% CI 0.478 to 0.689), and adding the score to age, sex, and stage did not improve discrimination (C-index 0.885 for the clinical model and 0.878 with the risk score added; likelihood-ratio *p* = 0.953).

### 3.6. Evaluation in Additional Independent Cohorts

With the same fixed coefficients and standardization, the risk score was not associated with outcome in the two muscle-invasive cystectomy cohorts, GSE31684 (HR per standard deviation 0.87, 95% CI 0.65 to 1.15; 65 deaths) and GSE48075 (1.14, 0.82 to 1.60; 45 deaths), in the mainly non-muscle-invasive cohort GSE32894 (1.24, 0.83 to 1.86; 25 disease-specific deaths), or in the atezolizumab-treated IMvigor210 cohort (0.91, 0.80 to 1.03; 232 deaths), with C-indices between 0.502 and 0.530 (Table S4). Adjustment for stage, where available, did not change these results. In the smaller ICI-treated cohort GSE176307, a higher risk score was associated with shorter overall survival (HR 1.37, 1.07 to 1.74, *p* = 0.011; C-index 0.583; 57 deaths). The score did not discriminate responders from non-responders in IMvigor210 (area under the curve 0.474, 95% CI 0.389 to 0.560) and did so weakly and imprecisely in GSE176307 (0.678, 0.504 to 0.852). Because both ICI-treated cohorts are single-arm, these analyses cannot distinguish a prognostic from a predictive effect.

### 3.7. Association Between the Seven-Gene Risk Score and Immune Indices

**Figure 4:**
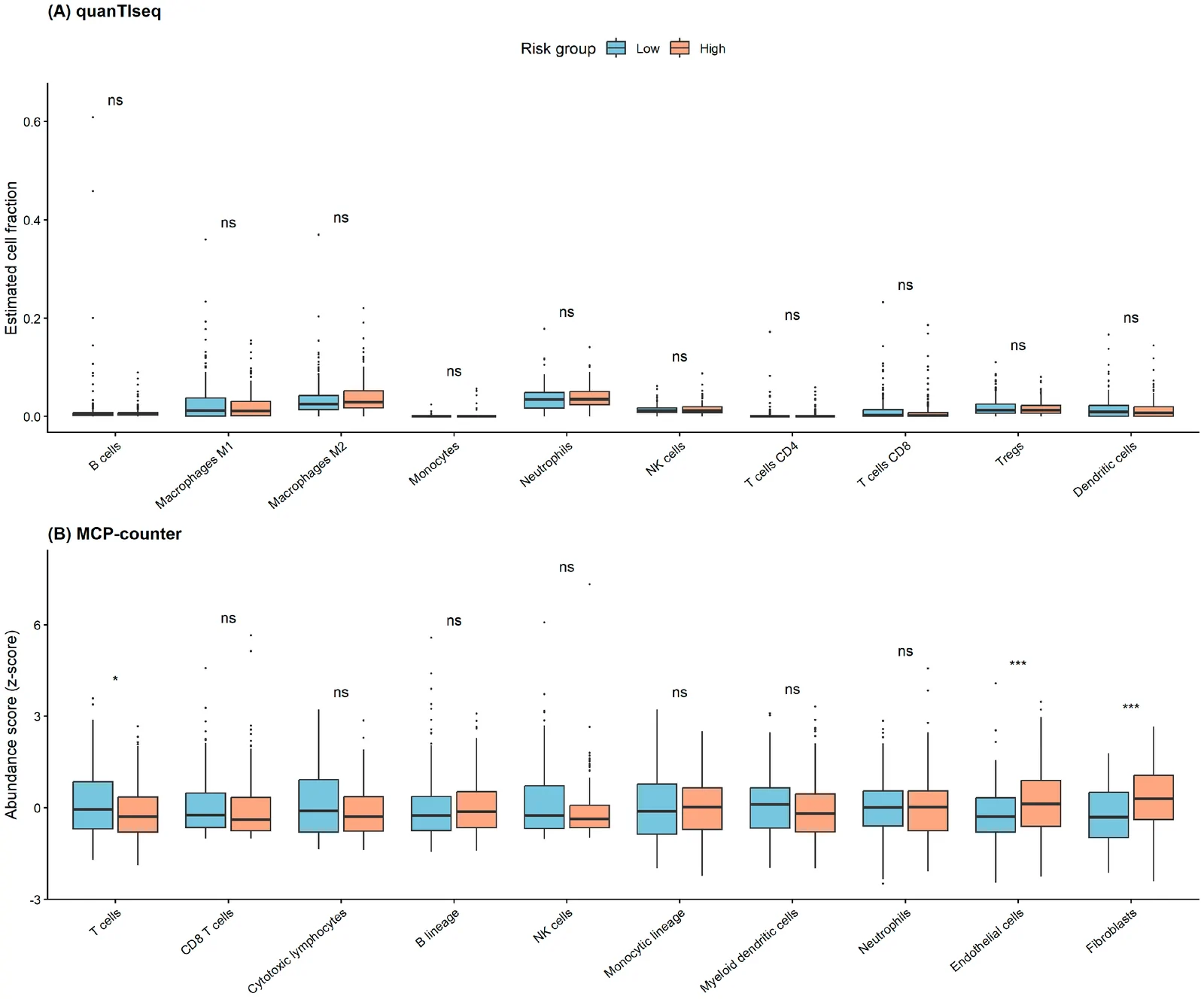
Transcriptome-inferred immune-cell composition of TCGA-BLCA tumors by risk group (*n* = 404). (**A**) Fractions of ten immune-cell types estimated with quanTIseq. (**B**) Abundance scores of eight immune and two stromal cell populations estimated with MCP-counter, shown as z-scores. Risk groups were defined at the cohort median. Boxes show the median and interquartile range, whiskers extend to 1.5 times the interquartile range, and dots indicate values beyond the whiskers. Groups were compared with the Wilcoxon rank-sum test, and q-values are Benjamini–Hochberg-adjusted within each method: * q < 0.05, *** q < 0.001; ns, not significant.

We next asked whether the risk score is associated with transcriptome-inferred indices of the tumor immune microenvironment in TCGA-BLCA. With quanTIseq, none of the ten immune-cell fractions differed significantly between risk groups after correction for multiple testing (all q > 0.26; Figure 4A). With MCP-counter, high-risk tumors had higher fibroblast (q < 0.001) and endothelial cell (q = 0.001) abundance scores and slightly lower T-cell scores (q = 0.046), whereas the other immune populations, including CD8 T cells (q = 0.216) and cytotoxic lymphocytes (q = 0.225), did not differ significantly (Figure 4B; Table S5).

**Figure 5:**
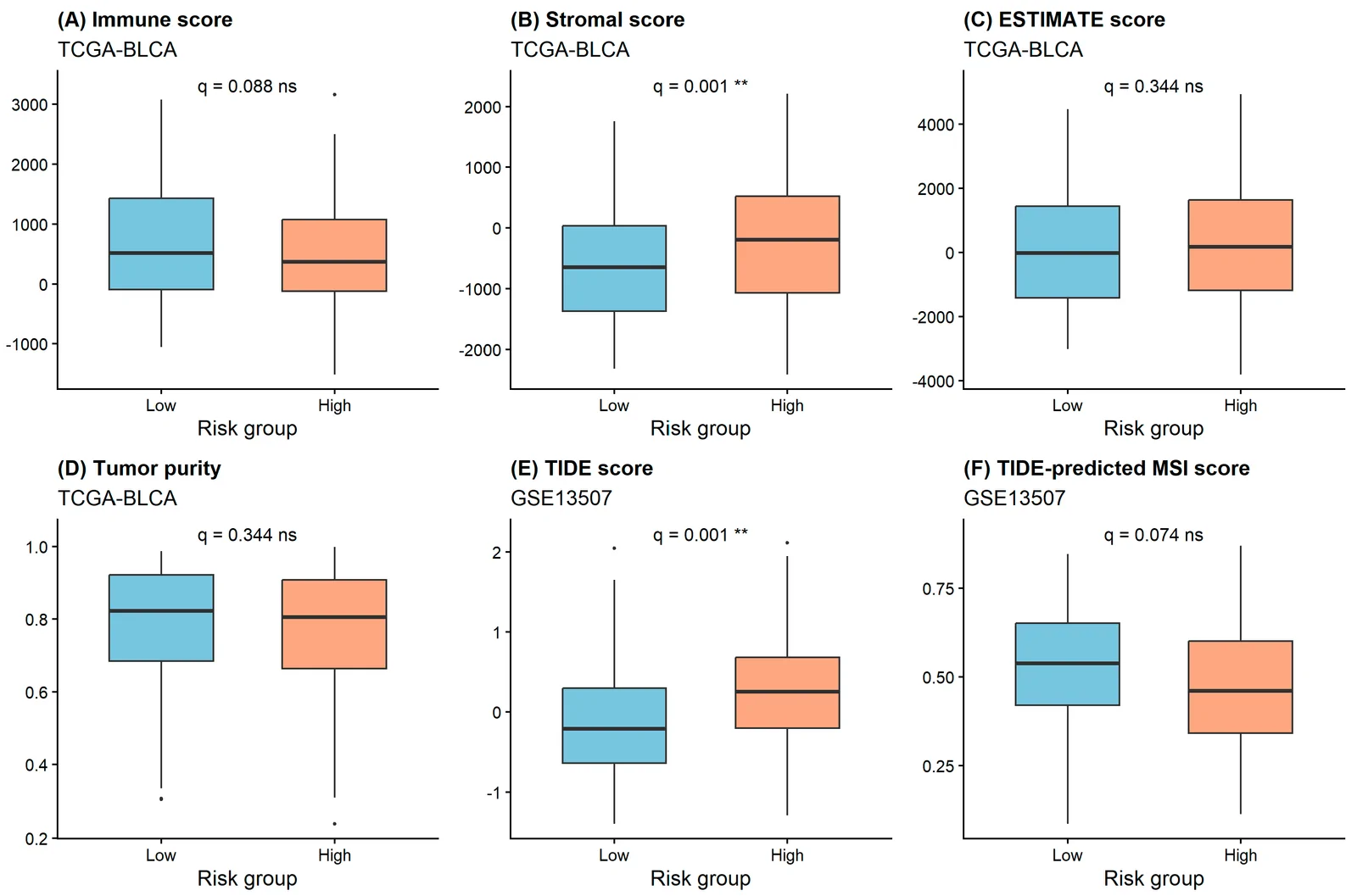
Transcriptome-inferred tumor microenvironment indices by risk group. (**A**) Immune score, (**B**) stromal score, (**C**) ESTIMATE score, and (**D**) tumor purity estimated with ESTIMATE in TCGA-BLCA (*n* = 404); (**E**) TIDE score and (**F**) TIDE-predicted microsatellite instability (MSI) score in GSE13507 (*n* = 165). Risk groups were defined at the cohort median. Boxes show the median and interquartile range, whiskers extend to 1.5 times the interquartile range, and dots indicate values beyond the whiskers. Groups were compared with the Wilcoxon rank-sum test, and q-values are Benjamini–Hochberg-adjusted across the six comparisons: ** q < 0. 01; ns, not significant.

We used ESTIMATE, which calculates immune and stromal scores to assess tumor purity [28]. In TCGA-BLCA, high-risk tumors had higher stromal scores than low-risk tumors (median −189 vs. −641; q = 0.001), whereas immune scores (374 vs. 522; q = 0.088), ESTIMATE scores (188 vs. −11; q = 0.344), and tumor purity (0.81 vs. 0.82; q = 0.344) did not differ significantly after correction for multiple testing (Figure 5A–D). We also conducted TIDE analysis, which evaluates T cell dysfunction and exclusion in tumors [29]. In GSE13507, high-risk tumors had higher TIDE scores (median 0.25 vs. −0.21; q = 0.001), whereas the TIDE-predicted microsatellite instability (MSI) score did not differ significantly (0.46 vs. 0.54; q = 0.074) (Figure 5E,F). MSI-high tumors are more sensitive to ICIs [45,46], but TIDE and TIDE-predicted MSI are computational surrogates and neither cohort received ICIs; these findings therefore do not indicate that the risk score predicts benefit from ICIs.

## 4. Discussion

PD-1 and its ligand PD-L1 suppress antitumor immune responses, particularly T-cell activation, proliferation, and cytotoxic function. Several mediators of the interaction between PD-1 and T cells have been identified, including glycosphingolipid-enriched microdomains-1 and interleukin-6 [47,48]. PD-1 signaling also interacts extensively with other pathways, including PI3K/AKT, MAPK, JAK-STAT, WNT, NF-κB, and Hedgehog signaling pathways, as well as specific microRNAs and lncRNAs [49]. Over the past decade, PD-1/PD-L1 inhibitors have changed clinical practice in oncology. In 2014, the anti-PD-1 antibody pembrolizumab became the first PD-1/PD-L1 inhibitor approved by the U.S. Food and Drug Administration for advanced melanoma [50]. Atezolizumab, an anti-PD-L1 antibody, was approved for advanced bladder cancer in 2016 [13]. However, objective response rates to ICIs in bladder cancer range only from 20% to 38% [10,12,51,52,53]. Biomarkers that identify patients likely to benefit are therefore needed. In addition to the biomarkers mentioned above, transcriptome-based molecular classification has provided insight into the biology of bladder cancer. For example, Kamoun et al. reported six molecular classes for muscle-invasive bladder cancer [54]. We therefore combined pathway-based gene selection with penalized Cox regression to derive a prognostic transcriptomic signature.

Cox proportional hazards regression is the standard method for identifying genes associated with survival. However, in high-throughput datasets, the number of genes far exceeds the number of samples. Penalized regression and other machine learning methods are therefore used to select prognostic features from such data [55]. Unlike most transcriptomic signatures in bladder cancer, which are derived from genome-wide screens, our panel was restricted a priori to genes annotated to PD-1 pathway biology, which keeps the signature small and interpretable. Using TCGA-BLCA as the training cohort, we derived a seven-gene risk score. The association of this panel with survival was then examined in an independent cohort, GSE13507, and in five additional public cohorts, including two treated with ICIs.

The seven-gene risk score comprises *FYN*, *GRAP2*, *TRIB3*, *MAP3K8*, *AKT3*, *CD274*, and *CD80*. FYN is a non-receptor tyrosine kinase involved in cell migration and in the development of bladder, breast, and pancreatic cancer [56,57]. Du et al. showed that KLF5 overexpression upregulates *FYN*, promoting bladder cancer cell migration and lamellipodia formation [58]. TRIB3 is a pseudokinase that regulates many physiological processes, such as cell proliferation, differentiation, metabolism, apoptosis, and stress. Yang et al. demonstrated that TRIB3 could promote the progression of bladder cancer [59]. TRIB3 has also been shown to reduce CD8+ T-cell infiltration and to promote immune evasion by repressing the STAT1-CXCL10 axis, and its ablation sensitized colorectal tumors to immune checkpoint blockade [60]; this is consistent with the positive coefficient of TRIB3 in our signature, although these observations were made outside bladder cancer. AKT3 regulates cell proliferation, and *AKT3* knockdown causes mitochondrial dysfunction in bladder cancer cells [61]. *CD274* encodes PD-L1. Interestingly, its LASSO coefficient was negative (Table 1), indicating that higher *CD274* mRNA expression was associated with better OS. Olkhov-Mitsel et al. showed that *CD274* mRNA expression correlates strongly with PD-L1 protein expression [62]. Because TCGA-BLCA samples were collected before ICIs entered clinical practice, this association cannot be attributed to treatment; a more plausible explanation is that *CD274* expression marks an inflamed, T cell-infiltrated microenvironment, which is itself associated with better outcomes in bladder cancer. According to the Human Protein Atlas (https://www.proteinatlas.org/), higher *CD274* mRNA expression is also associated with favorable prognosis in colorectal and breast cancer. CD80 (B7-1) is a ligand of CD28 and provides co-stimulatory signals for T-cell activation [63].

Little is known about the roles of GRAP2 and MAP3K8 in bladder cancer. GRAP2 was identified as a binding partner of Rad, a Ras-related GTPase that promotes cell growth. Lee et al. reported that Rad might induce carcinogenesis [64]. Activation of MAP3K8, also known as TPL2, has been associated with the proliferation and progression of colorectal cancer [65,66], renal cell carcinoma [67], prostate cancer [68], and breast cancer [69]. However, MAP3K8 may also act as a tumor suppressor in several malignancies [70,71,72], and the effect of MAP3K8 on bladder cancer is still unclear. In the immune system, MAP3K8 is required for TNF-α production by macrophages [73] and constrains FoxP3 expression and the suppressive function of regulatory T cells [74], although Tpl2 ablation has also been reported to reduce regulatory T-cell generation in an intestinal tumor model [72], and its role in tumor immunity appears context-dependent [75]. A pro-inflammatory function of MAP3K8 would be consistent with its negative coefficient in our signature, but our data do not establish a mechanistic link for either gene.

For clinical use, a signature must add prognostic information to routinely available variables rather than merely be associated with survival. In TCGA-BLCA, adding the risk score to age, sex, and stage increased the C-index from 0.630 to 0.664, with good calibration after bootstrap correction for optimism. This incremental gain is modest and of a magnitude similar to that reported for other transcriptomic signatures in bladder cancer. Many such signatures have been derived from TCGA-BLCA with LASSO-Cox regression and immune-related candidate genes [76,77]; training-cohort time-dependent AUCs of about 0.71–0.77 have been reported [76], whereas a multi-cohort study reported a mean C-index of 0.658 across four validation cohorts [78]. Signatures derived from the same data with similar methods often share few genes [76,77], and many published prognostic signatures perform no better than random gene sets of similar size [79] or show unstable gene lists across training sets [80]. Our bootstrap analysis showed the same instability: *TRIB3*, *MAP3K8*, and *CD274* were selected consistently, whereas the selection of the remaining genes depended on the resample. The signature should therefore be regarded as exploratory.

The most important finding of this study, however, is negative. When the coefficients and standardization were fixed in advance, the association observed in TCGA-BLCA was not reproduced in GSE13507 or in the three other independent cohorts without ICI treatment, including two muscle-invasive cystectomy cohorts in which the clinical setting most closely resembles that of TCGA-BLCA. Differences in platform, endpoint, and case mix may contribute, but the modest effect size in the training cohort and the instability of gene selection under bootstrap resampling suggest that much of the training-cohort association reflects optimism that does not transport to new data [79,80]. The positive association in the small GSE176307 cohort, which was not observed in the larger IMvigor210 cohort, should be regarded as a possible chance finding until replicated. In GSE13507, the univariate and multivariate results were concordant, with both being null, and stage was by far the strongest prognostic factor. Taken together, these findings indicate that the signature, as currently specified, should not be used for prognostication outside research settings.

Circulating tumor DNA (ctDNA) is an emerging prognostic and potentially predictive marker in muscle-invasive bladder cancer. ctDNA detects molecular residual disease after cystectomy, often months before radiographic relapse [81]. In an exploratory analysis of IMvigor010, ctDNA-positive patients had a worse prognosis (hazard ratio 6.3 in the observation arm) and derived disease-free and overall survival benefit from adjuvant atezolizumab, whereas ctDNA-negative patients did not [82], and the phase 3 IMvigor011 trial subsequently showed that ctDNA-guided adjuvant atezolizumab prolonged disease-free and overall survival in ctDNA-positive patients [83]. A tissue-based transcriptomic signature measures a different dimension, namely tumor-intrinsic and microenvironmental biology at the time of resection, whereas ctDNA reflects residual disease burden. The two could therefore be complementary, for example in ctDNA-negative patients or where serial ctDNA testing is not available, but this possibility is hypothetical and would need to be tested in cohorts with both measurements. A seven-gene signature is in principle suitable for a targeted RT-qPCR assay, as has been done for other multigene prognostic assays [84], but this would require re-estimating and locking the coefficients on the new platform and validating the assay in independent cohorts. To facilitate independent evaluation, the coefficients of the final model are provided in Table 1 and the standardization procedure is described in Section 2.4.

Two further points concern interpretation. First, TCGA-BLCA contains very few stage I tumors (2 of 404), so stage was dichotomized as I/II versus III/IV in the multivariable models; this grouping avoids unstable estimates for stage I and corresponds to the clinically relevant distinction between organ-confined and locally advanced or node-positive disease. Second, the immune analyses should be interpreted with caution: the signature was derived from PD-1 pathway genes, and quanTIseq, MCP-counter, ESTIMATE, and the Reactome and KEGG annotations draw on the same or closely related transcriptomic information. The associations between the risk score and these indices therefore represent concordance between related transcriptomic features rather than independent biological or mechanistic validation. In the re-analysis, the main microenvironmental differences were higher stromal scores and higher fibroblast and endothelial cell abundance in high-risk tumors, together with higher TIDE scores in GSE13507, whereas differences in immune scores, tumor purity, and immune-cell fractions were small or not significant after correction for multiple testing; the data therefore point to a stroma-rich rather than a simply immunologically cold phenotype.

This study has several limitations. First, it is an in silico analysis of retrospective public datasets, and the signature has not been examined experimentally or in a prospective series. Second, the original model was derived from a DriverDB version of TCGA-BLCA whose intermediate files are no longer available; all results in this version were re-derived from public data, and the original LASSO fit, including its random seed, could not be re-created exactly. Third, the external cohorts differ from TCGA-BLCA in platform, endpoint, and case mix, and several are small; these differences may have contributed to the failure to replicate, but they also reflect the heterogeneity across which a clinically useful signature would need to perform. Fourth, the composition of the panel was sensitive to resampling, and restricting candidates to genes differentially expressed between tumor and normal tissue, with only 19 normal samples, may have excluded prognostic genes without tumor-normal differences. Fifth, the cohorts consist predominantly of urothelial carcinoma, so the signature has not been evaluated in other histologies. Sixth, immune-cell fractions, stromal and immune scores, tumor purity, and TIDE scores were estimated computationally from transcriptomic data rather than measured directly by immunohistochemistry or immunofluorescence. Finally, the ICI-treated cohorts were single-arm, so no conclusion can be drawn about whether the signature predicts benefit from ICIs; that would require randomized cohorts and a treatment-by-biomarker interaction analysis.

## 5. Conclusions

In conclusion, we derived a seven-gene risk score from PD-1 pathway genes that was associated with overall survival in TCGA-BLCA independently of age, sex, and stage and that modestly improved discrimination over clinical variables. However, with its coefficients and standardization fixed in advance, the score was not associated with outcome in GSE13507 or in four of five additional independent cohorts, and its gene composition was unstable under resampling. The signature should therefore be regarded as exploratory. These results underline the need to evaluate pathway-restricted transcriptomic signatures with prespecified procedures in multiple independent muscle-invasive cohorts, and in randomized ICI-treated cohorts for any predictive claim, before clinical use.

## Figures and Tables

**Table 1:** Genes in the seven-gene risk score, with log_2_ fold change and Benjamini–Hochberg-adjusted *p*-value for tumor vs. normal tissue (DESeq2) and LASSO coefficient.

Genes	Log_2_ Fold Change	Adjusted *p* Value	LASSO Coefficient
*FYN*	−1.4975	1.83 × 10^−7^	0.957
*GRAP2*	−1.2311	0.0021	−0.134
*TRIB3*	1.6772	3.40 × 10^−8^	1.298
*MAP3K8*	−1.4034	3.84 × 10^−9^	−0.844
*AKT3*	−1.9588	8.50 × 10^−9^	0.498
*CD274*	1.1764	0.0175	−0.936
*CD80*	1.0874	0.0264	−0.644

Abbreviations: LASSO, least absolute shrinkage and selection operator.

**Table 2:** Patient characteristics by risk group in TCGA-BLCA and GSE13507. *p* values were calculated with the Wilcoxon rank-sum test (age) and Fisher’s exact test (sex and stage) and are not adjusted for multiple comparisons. Stage was unknown for two low-risk patients in TCGA-BLCA.

	Training Cohort (TCGA-BLCA) *n* = 404			External Evaluation Cohort (GSE13507) *n* = 165		
	High Risk	Low Risk	*p* Value	High Risk	Low Risk	*p* Value
Age (years), median (IQR)	70 (62–78)	67 (59–75)	0.021	66 (60–73)	67 (59–74)	0.778
Sex, *n* (%)			0.910			1.000
Female	54 (26.7)	52 (25.7)		15 (18.3)	15 (18.1)	
Male	148 (73.3)	150 (74.3)		67 (81.7)	68 (81.9)	
Stage, *n* (%)			0.003			0.010
I	1 (0.5)	1 (0.5)		46 (56.1)	57 (68.7)	
II	48 (23.8)	80 (39.6)		16 (19.5)	10 (12.0)	
III	76 (37.6)	64 (31.7)		12 (14.6)	2 (2.4)	
IV	77 (38.1)	55 (27.2)		8 (9.8)	14 (16.9)	

Abbreviations: IQR, interquartile range.

**Table 3:** Univariate and multivariate Cox regression analysis for overall survival in TCGA-BLCA (404 patients, 177 deaths; multivariate model, 402 patients with complete data, 176 deaths).

	Univariate			Multivariate		
Variables	HR	95% CI	*p* Value	HR	95% CI	*p* Value
Risk score						
High	1.97	1.45–2.66	<0.001	1.75	1.28–2.39	<0.001
Low	1.00			1.00		
Age						
≥65 years	2.02	1.44–2.84	<0.001	1.84	1.31–2.60	<0.001
<65 years	1.00			1.00		
Sex						
Male	0.90	0.65–1.24	0.507	0.81	0.58–1.13	0.218
Female	1.00			1.00		
Stage						
III + IV	2.16	1.49–3.13	<0.001	1.91	1.31–2.78	<0.001
I + II	1.00			1.00		

Abbreviations: CI, confidence interval; HR, hazard ratio; OS, overall survival.

**Table 4:** Univariate and multivariate Cox regression analysis for cancer-specific survival in GSE13507 (165 patients, 32 cancer-specific deaths).

	Univariate			Multivariate		
Variables	HR	95% CI	*p* Value	HR	95% CI	*p* Value
Risk score						
High	1.28	0.64–2.58	0.486	0.96	0.47–1.96	0.905
Low	1.00			1.00		
Age						
≥65 years	3.11	1.34–7.21	0.008	3.29	1.40–7.74	0.006
<65 years	1.00			1.00		
Sex						
Male	0.48	0.22–1.03	0.060	0.56	0.26–1.21	0.140
Female	1.00			1.00		
Stage						
II + III + IV	24.7	7.51–81.5	<0.001	25.9	7.82–86.0	<0.001
I	1.00			1.00		

Abbreviations: CI, confidence interval; HR, hazard ratio; CSS, cancer-specific survival.

## Data Availability

The datasets analyzed in this study are publicly available from the Genomic Data Commons data portal (TCGA-BLCA), DriverDB (https://driverdb.bioinfomics.org/, accessed on 12 October 2026), the Gene Expression Omnibus (GSE13507, GSE31684, GSE48075, GSE32894, and GSE176307), and the IMvigor210CoreBiologies R package (IMvigor210). The code used for all analyses in this version is provided in the Supplementary Materials (Code S1).

## Supplementary Materials

The following supporting information can be downloaded at: https://www.mdpi.com/article/10.3390/cimb48101056/s1.

## References

[1] Bray F., Laversanne M., Sung H., Ferlay J., Siegel R.L., Soerjomataram I., Jemal A. (2024). Global cancer statistics 2022: GLOBOCAN estimates of incidence and mortality worldwide for 36 cancers in 185 countries. CA Cancer J. Clin..

[2] Cumberbatch M.G.K., Jubber I., Black P.C., Esperto F., Figueroa J.D., Kamat A.M., Kiemeney L., Lotan Y., Pang K., Silverman D.T. (2018). Epidemiology of Bladder Cancer: A Systematic Review and Contemporary Update of Risk Factors in 2018. Eur. Urol..

[3] Jung I., Messing E. (2000). Molecular mechanisms and pathways in bladder cancer development and progression. Cancer Control.

[4] Kogevinas M., ’t Mannetje A., Cordier S., Ranft U., Gonzalez C.A., Vineis P., Chang-Claude J., Lynge E., Wahrendorf J., Tzonou A. (2003). Occupation and bladder cancer among men in Western Europe. Cancer Causes Control.

[5] Golijanin D.J., Kakiashvili D., Madeb R.R., Messing E.M., Lerner S.P. (2006). Chemoprevention of bladder cancer. World J. Urol..

[6] Patel V.G., Oh W.K., Galsky M.D. (2020). Treatment of muscle-invasive and advanced bladder cancer in 2020. CA Cancer J. Clin..

[7] Bamias A., Dafni U., Karadimou A., Timotheadou E., Aravantinos G., Psyrri A., Xanthakis I., Tsiatas M., Koutoulidis V., Constantinidis C. (2013). Prospective, open-label, randomized, phase III study of two dose-dense regimens MVAC versus gemcitabine/cisplatin in patients with inoperable, metastatic or relapsed urothelial cancer: A Hellenic Cooperative Oncology Group study (HE 16/03). Ann. Oncol..

[8] von der Maase H., Sengelov L., Roberts J.T., Ricci S., Dogliotti L., Oliver T., Moore M.J., Zimmermann A., Arning M. (2005). Long-term survival results of a randomized trial comparing gemcitabine plus cisplatin, with methotrexate, vinblastine, doxorubicin, plus cisplatin in patients with bladder cancer. J. Clin. Oncol..

[9] Balar A.V., Castellano D., O’Donnell P.H., Grivas P., Vuky J., Powles T., Plimack E.R., Hahn N.M., de Wit R., Pang L. (2017). First-line pembrolizumab in cisplatin-ineligible patients with locally advanced and unresectable or metastatic urothelial cancer (KEYNOTE-052): A multicentre, single-arm, phase 2 study. Lancet Oncol..

[10] Bellmunt J., de Wit R., Vaughn D.J., Fradet Y., Lee J.L., Fong L., Vogelzang N.J., Climent M.A., Petrylak D.P., Choueiri T.K. (2017). Keynote- Investigators Pembrolizumab as Second-Line Therapy for Advanced Urothelial Carcinoma. N. Engl. J. Med..

[11] Sharma P., Callahan M.K., Bono P., Kim J., Spiliopoulou P., Calvo E., Pillai R.N., Ott P.A., de Braud F., Morse M. (2016). Nivolumab monotherapy in recurrent metastatic urothelial carcinoma (CheckMate 032): A multicentre, open-label, two-stage, multi-arm, phase 1/2 trial. Lancet Oncol..

[12] Balar A.V., Galsky M.D., Rosenberg J.E., Powles T., Petrylak D.P., Bellmunt J., Loriot Y., Necchi A., Hoffman-Censits J., Perez-Gracia J.L. (2017). Atezolizumab as first-line treatment in cisplatin-ineligible patients with locally advanced and metastatic urothelial carcinoma: A single-arm, multicentre, phase 2 trial. Lancet.

[13] Rosenberg J.E., Hoffman-Censits J., Powles T., van der Heijden M.S., Balar A.V., Necchi A., Dawson N., O’Donnell P.H., Balmanoukian A., Loriot Y. (2016). Atezolizumab in patients with locally advanced and metastatic urothelial carcinoma who have progressed following treatment with platinum-based chemotherapy: A single-arm, multicentre, phase 2 trial. Lancet.

[14] Apolo A.B., Infante J.R., Balmanoukian A., Patel M.R., Wang D., Kelly K., Mega A.E., Britten C.D., Ravaud A., Mita A.C. (2017). Avelumab, an Anti-Programmed Death-Ligand 1 Antibody, In Patients with Refractory Metastatic Urothelial Carcinoma: Results from a Multicenter, Phase Ib Study. J. Clin. Oncol..

[15] Patel M.R., Ellerton J., Infante J.R., Agrawal M., Gordon M., Aljumaily R., Britten C.D., Dirix L., Lee K.-W., Taylor M. (2018). Avelumab in metastatic urothelial carcinoma after platinum failure (JAVELIN Solid Tumor): Pooled results from two expansion cohorts of an open-label, phase 1 trial. Lancet Oncol..

[16] Powles T., O’Donnell P.H., Massard C., Arkenau H.T., Friedlander T.W., Hoimes C.J., Lee J.L., Ong M., Sridhar S.S., Vogelzang N.J. (2017). Efficacy and Safety of Durvalumab in Locally Advanced or Metastatic Urothelial Carcinoma: Updated Results from a Phase 1/2 Open-label Study. JAMA Oncol..

[17] Sharma P., Siefker-Radtke A., de Braud F., Basso U., Calvo E., Bono P., Morse M.A., Ascierto P.A., Lopez-Martin J., Brossart P. (2019). Nivolumab Alone and With Ipilimumab in Previously Treated Metastatic Urothelial Carcinoma: CheckMate 032 Nivolumab 1 mg/kg Plus Ipilimumab 3 mg/kg Expansion Cohort Results. J. Clin. Oncol..

[18] Powles T., Csőszi T., Özgüroğlu M., Matsubara N., Géczi L., Cheng S.Y.S., Fradet Y., Oudard S., Vulsteke C., Morales Barrera R. (2021). Pembrolizumab alone or combined with chemotherapy versus chemotherapy as first-line therapy for advanced urothelial carcinoma (KEYNOTE-361): A randomised, open-label, phase 3 trial. Lancet Oncol..

[19] Powles T., van der Heijden M.S., Castellano D., Galsky M.D., Loriot Y., Petrylak D.P., Ogawa O., Park S.H., Lee J.-L., De Giorgi U. (2020). Durvalumab alone and durvalumab plus tremelimumab versus chemotherapy in previously untreated patients with unresectable, locally advanced or metastatic urothelial carcinoma (DANUBE): A randomised, open-label, multicentre, phase 3 trial. Lancet Oncol..

[20] Powles T., Valderrama B.P., Gupta S., Bedke J., Kikuchi E., Hoffman-Censits J., Iyer G., Vulsteke C., Park S.H., Shin S.J. (2024). Enfortumab Vedotin and Pembrolizumab in Untreated Advanced Urothelial Cancer. N. Engl. J. Med..

[21] Tamalunas A., Aydogdu C., Unterrainer L.M., Schott M., Rodler S., Ledderose S., Schulz G.B., Stief C.G., Casuscelli J. (2024). The Vanishing Clinical Value of PD-L1 Status as a Predictive Biomarker in the First-Line Treatment of Urothelial Carcinoma of the Bladder. Cancers.

[22] Sanguedolce F., Falagario U.G., Zanelli M., Palicelli A., Zizzo M., Busetto G.M., Cormio A., Carrieri G., Cormio L. (2024). Integrating the PD-L1 Prognostic Biomarker in Non-Muscle Invasive Bladder Cancer in Clinical Practice-A Comprehensive Review on State-of-the-Art Advances and Critical Issues. J. Clin. Med..

[23] Akhtar M., Al-Bozom I.A., Al Hussain T. (2019). Papillary Renal Cell Carcinoma (PRCC): An Update. Adv. Anat. Pathol..

[24] Yarchoan M., Hopkins A., Jaffee E.M. (2017). Tumor Mutational Burden and Response Rate to PD-1 Inhibition. N. Engl. J. Med..

[25] Mariathasan S., Turley S.J., Nickles D., Castiglioni A., Yuen K., Wang Y., Kadel E.E., Koeppen H., Astarita J.L., Cubas R. (2018). TGFbeta attenuates tumour response to PD-L1 blockade by contributing to exclusion of T cells. Nature.

[26] Ghatalia P., Plimack E. (2019). Biomarkers for neoadjuvant checkpoint blockade response in urothelial cancer. Nat. Med..

[27] Paijens S.T., Vledder A., de Bruyn M., Nijman H.W. (2021). Tumor-infiltrating lymphocytes in the immunotherapy era. Cell. Mol. Immunol..

[28] Yoshihara K., Shahmoradgoli M., Martinez E., Vegesna R., Kim H., Torres-Garcia W., Trevino V., Shen H., Laird P.W., Levine D.A. (2013). Inferring tumour purity and stromal and immune cell admixture from expression data. Nat. Commun..

[29] Jiang P., Gu S., Pan D., Fu J., Sahu A., Hu X., Li Z., Traugh N., Bu X., Li B. (2018). Signatures of T cell dysfunction and exclusion predict cancer immunotherapy response. Nat. Med..

[30] Cheng W.C., Chung I.F., Chen C.Y., Sun H.J., Fen J.J., Tang W.C., Chang T.Y., Wong T.T., Wang H.W. (2014). DriverDB: An exome sequencing database for cancer driver gene identification. Nucleic Acids Res..

[31] Chung I.F., Chen C.Y., Su S.C., Li C.Y., Wu K.J., Wang H.W., Cheng W.C. (2016). DriverDBv2: A database for human cancer driver gene research. Nucleic Acids Res..

[32] Liu S.H., Shen P.C., Chen C.Y., Hsu A.N., Cho Y.C., Lai Y.L., Chen F.H., Li C.Y., Wang S.C., Chen M. (2020). DriverDBv3: A multi-omics database for cancer driver gene research. Nucleic Acids Res..

[33] Liu C.H., Lai Y.L., Shen P.C., Liu H.C., Tsai M.H., Wang Y.D., Lin W.J., Chen F.H., Li C.Y., Wang S.C. (2024). DriverDBv4: A multi-omics integration database for cancer driver gene research. Nucleic Acids Res..

[34] Liu J., Lichtenberg T., Hoadley K.A., Poisson L.M., Lazar A.J., Cherniack A.D., Kovatich A.J., Benz C.C., Levine D.A., Lee A.V. (2018). An Integrated TCGA Pan-Cancer Clinical Data Resource to Drive High-Quality Survival Outcome Analytics. Cell.

[35] Fabregat A., Sidiropoulos K., Viteri G., Forner O., Marin-Garcia P., Arnau V., D’Eustachio P., Stein L., Hermjakob H. (2017). Reactome pathway analysis: A high-performance in-memory approach. BMC Bioinform..

[36] Love M.I., Huber W., Anders S. (2014). Moderated estimation of fold change and dispersion for RNA-seq data with DESeq2. Genome Biol..

[37] Friedman J., Hastie T., Tibshirani R. (2010). Regularization Paths for Generalized Linear Models via Coordinate Descent. J. Stat. Softw..

[38] Simon N., Friedman J., Hastie T., Tibshirani R. (2011). Regularization Paths for Cox’s Proportional Hazards Model via Coordinate Descent. J. Stat. Softw..

[39] Lee J.S., Leem S.H., Lee S.Y., Kim S.C., Park E.S., Kim S.B., Kim S.K., Kim Y.J., Kim W.J., Chu I.S. (2010). Expression signature of E2F1 and its associated genes predict superficial to invasive progression of bladder tumors. J. Clin. Oncol..

[40] Newman A.M., Liu C.L., Green M.R., Gentles A.J., Feng W., Xu Y., Hoang C.D., Diehn M., Alizadeh A.A. (2015). Robust enumeration of cell subsets from tissue expression profiles. Nat. Methods.

[41] Finotello F., Mayer C., Plattner C., Laschober G., Rieder D., Hackl H., Krogsdam A., Loncova Z., Posch W., Wilflingseder D. (2019). Molecular and pharmacological modulators of the tumor immune contexture revealed by deconvolution of RNA-seq data. Genome Med..

[42] Becht E., Giraldo N.A., Lacroix L., Buttard B., Elarouci N., Petitprez F., Selves J., Laurent-Puig P., Sautès-Fridman C., Fridman W.H. (2016). Estimating the population abundance of tissue-infiltrating immune and stromal cell populations using gene expression. Genome Biol..

[43] The Cancer Genome Atlas Research Network (2014). Comprehensive molecular characterization of urothelial bladder carcinoma. Nature.

[44] Kanehisa M., Sato Y., Kawashima M., Furumichi M., Tanabe M. (2016). KEGG as a reference resource for gene and protein annotation. Nucleic Acids Res..

[45] Chang L., Chang M., Chang H.M., Chang F. (2018). Microsatellite Instability: A Predictive Biomarker for Cancer Immunotherapy. Appl. Immunohistochem. Mol. Morphol..

[46] Petrelli F., Ghidini M., Ghidini A., Tomasello G. (2020). Outcomes Following Immune Checkpoint Inhibitor Treatment of Patients With Microsatellite Instability-High Cancers: A Systematic Review and Meta-analysis. JAMA Oncol..

[47] Strazza M., Azoulay-Alfaguter I., Peled M., Adam K., Mor A. (2021). Transmembrane adaptor protein PAG is a mediator of PD-1 inhibitory signaling in human T cells. Commun. Biol..

[48] Tanaka R., Ichimura Y., Kubota N., Saito A., Nakamura Y., Ishitsuka Y., Watanabe R., Fujisawa Y., Kanzaki M., Mizuno S. (2020). Activation of CD8 T cells accelerates anti-PD-1 antibody-induced psoriasis-like dermatitis through IL-6. Commun. Biol..

[49] Lin X., Kang K., Chen P., Zeng Z., Li G., Xiong W., Yi M., Xiang B. (2024). Regulatory mechanisms of PD-1/PD-L1 in cancers. Mol. Cancer.

[50] Robert C., Ribas A., Wolchok J.D., Hodi F.S., Hamid O., Kefford R., Weber J.S., Joshua A.M., Hwu W.-J., Gangadhar T.C. (2014). Anti-programmed-death-receptor-1 treatment with pembrolizumab in ipilimumab-refractory advanced melanoma: A randomised dose-comparison cohort of a phase 1 trial. Lancet.

[51] Fradet Y., Bellmunt J., Vaughn D.J., Lee J.L., Fong L., Vogelzang N.J., Climent M.A., Petrylak D.P., Choueiri T.K., Necchi A. (2019). Randomized phase III KEYNOTE-045 trial of pembrolizumab versus paclitaxel, docetaxel, or vinflunine in recurrent advanced urothelial cancer: Results of >2 years of follow-up. Ann. Oncol..

[52] Sharma P., Retz M., Siefker-Radtke A., Baron A., Necchi A., Bedke J., Plimack E.R., Vaena D., Grimm M.O., Bracarda S. (2017). Nivolumab in metastatic urothelial carcinoma after platinum therapy (CheckMate 275): A multicentre, single-arm, phase 2 trial. Lancet Oncol..

[53] Vuky J., Balar A.V., Castellano D., O’Donnell P.H., Grivas P., Bellmunt J., Powles T., Bajorin D., Hahn N.M., Savage M.J. (2020). Long-Term Outcomes in KEYNOTE-052: Phase II Study Investigating First-Line Pembrolizumab in Cisplatin-Ineligible Patients with Locally Advanced or Metastatic Urothelial Cancer. J. Clin. Oncol..

[54] Kamoun A., de Reynies A., Allory Y., Sjodahl G., Robertson A.G., Seiler R., Hoadley K.A., Groeneveld C.S., Al-Ahmadie H., Choi W. (2020). A Consensus Molecular Classification of Muscle-invasive Bladder Cancer. Eur. Urol..

[55] Kourou K., Exarchos T.P., Exarchos K.P., Karamouzis M.V., Fotiadis D.I. (2015). Machine learning applications in cancer prognosis and prediction. Comput. Struct. Biotechnol. J..

[56] Dong W., Sun S.J., Qin J.J., Liu G.M. (2020). Fyn stimulates the progression of pancreatic cancer via Fyn-GluN2b-AKT axis. Eur. Rev. Med. Pharmacol. Sci..

[57] Lee G.H., Yoo K.C., An Y., Lee H.J., Lee M., Uddin N., Kim M.J., Kim I.G., Suh Y., Lee S.J. (2018). FYN promotes mesenchymal phenotypes of basal type breast cancer cells through STAT5/NOTCH2 signaling node. Oncogene.

[58] Du C., Gao Y., Xu S., Jia J., Huang Z., Fan J., Wang X., He D., Guo P. (2016). KLF5 promotes cell migration by up-regulating FYN in bladder cancer cells. FEBS Lett..

[59] Yang J., Lin J., An J., Zhao Y., Jing S., Yu M., Zhu Y., Yao Y. (2021). TRIB3 Promotes the Malignant Progression of Bladder Cancer: An Integrated Analysis of Bioinformatics and in vitro Experiments. Front. Genet..

[60] Shang S., Yang Y.W., Chen F., Yu L., Shen S.H., Li K., Cui B., Lv X.X., Zhang C., Yang C. (2022). TRIB3 reduces CD8+ T cell infiltration and induces immune evasion by repressing the STAT1-CXCL10 axis in colorectal cancer. Sci. Transl. Med..

[61] Kim M., Kim Y.Y., Jee H.J., Bae S.S., Jeong N.Y., Um J.H., Yun J. (2016). Akt3 knockdown induces mitochondrial dysfunction in human cancer cells. Acta Biochim. Biophys. Sin..

[62] Olkhov-Mitsel E., Hodgson A., Liu S.K., Vesprini D., Bayani J., Bartlett J., Xu B., Downes M.R. (2021). Immune gene expression profiles in high-grade urothelial carcinoma of the bladder: A NanoString study. J. Clin. Pathol..

[63] Sansom D.M., Manzotti C.N., Zheng Y. (2003). What’s the difference between CD80 and CD86?. Trends Immunol..

[64] Lee I., Yeom S.Y., Lee S.J., Kang W.K., Park C. (2010). A novel senescence-evasion mechanism involving Grap2 and Cyclin D interacting protein inactivation by Ras associated with diabetes in cancer cells under doxorubicin treatment. Cancer Res..

[65] Tunca B., Tezcan G., Cecener G., Egeli U., Zorluoglu A., Yilmazlar T., Ak S., Yerci O., Ozturk E., Umut G. (2013). Overexpression of CK20, MAP3K8 and EIF5A correlates with poor prognosis in early-onset colorectal cancer patients. J. Cancer Res. Clin. Oncol..

[66] Wei R., Yang Q., Han B., Li Y., Yao K., Yang X., Chen Z., Yang S., Zhou J., Li M. (2017). microRNA-375 inhibits colorectal cancer cells proliferation by downregulating JAK2/STAT3 and MAP3K8/ERK signaling pathways. Oncotarget.

[67] Liu F., Chen N., Xiao R., Wang W., Pan Z. (2016). miR-144-3p serves as a tumor suppressor for renal cell carcinoma and inhibits its invasion and metastasis by targeting MAP3K8. Biochem. Biophys. Res. Commun..

[68] Kwon W., Choi S.K., Kim D., Kim H.G., Park J.K., Han J.E., Cho G.J., Yun S., Yu W., Han S.H. (2021). ZNF507 affects TGF-beta signaling via TGFBR1 and MAP3K8 activation in the progression of prostate cancer to an aggressive state. J. Exp. Clin. Cancer Res..

[69] Kim K., Kim G., Kim J.Y., Yun H.J., Lim S.C., Choi H.S. (2014). Interleukin-22 promotes epithelial cell transformation and breast tumorigenesis via MAP3K8 activation. Carcinogenesis.

[70] Gkirtzimanaki K., Gkouskou K.K., Oleksiewicz U., Nikolaidis G., Vyrla D., Liontos M., Pelekanou V., Kanellis D.C., Evangelou K., Stathopoulos E.N. (2013). TPL2 kinase is a suppressor of lung carcinogenesis. Proc. Natl. Acad. Sci. USA.

[71] Koliaraki V., Roulis M., Kollias G. (2012). Tpl2 regulates intestinal myofibroblast HGF release to suppress colitis-associated tumorigenesis. J. Clin. Investig..

[72] Serebrennikova O.B., Tsatsanis C., Mao C., Gounaris E., Ren W., Siracusa L.D., Eliopoulos A.G., Khazaie K., Tsichlis P.N. (2012). Tpl2 ablation promotes intestinal inflammation and tumorigenesis in Apcmin mice by inhibiting IL-10 secretion and regulatory T-cell generation. Proc. Natl. Acad. Sci. USA.

[73] Dumitru C.D., Ceci J.D., Tsatsanis C., Kontoyiannis D., Stamatakis K., Lin J.H., Patriotis C., Jenkins N.A., Copeland N.G., Kollias G. (2000). TNF-alpha induction by LPS is regulated posttranscriptionally via a Tpl2/ERK-dependent pathway. Cell.

[74] Li X., Acuff N.V., Peeks A.R., Kirkland R., Wyatt K.D., Nagy T., Watford W.T. (2016). Tumor Progression Locus 2 (Tpl2) Activates the Mammalian Target of Rapamycin (mTOR) Pathway, Inhibits Forkhead Box P3 (FoxP3) Expression, and Limits Regulatory T Cell (Treg) Immunosuppressive Functions. J. Biol. Chem..

[75] Njunge L.W., Estania A.P., Guo Y., Liu W., Yang L. (2020). Tumor progression locus 2 (TPL2) in tumor-promoting Inflammation, Tumorigenesis and Tumor Immunity. Theranostics.

[76] Qiu H., Hu X., He C., Yu B., Li Y., Li J. (2020). Identification and Validation of an Individualized Prognostic Signature of Bladder Cancer Based on Seven Immune Related Genes. Front. Genet..

[77] Luo Y., Chen L., Zhou Q., Xiong Y., Wang G., Liu X., Xiao Y., Ju L., Wang X. (2020). Identification of a prognostic gene signature based on an immunogenomic landscape analysis of bladder cancer. J. Cell. Mol. Med..

[78] Huang H., Shao H., Wang Y., Ge L. (2025). Disulfide bond-related gene signature development for bladder cancer prognosis prediction and immune microenvironment characterization. Sci. Rep..

[79] Venet D., Dumont J.E., Detours V. (2011). Most random gene expression signatures are significantly associated with breast cancer outcome. PLoS Comput. Biol..

[80] Michiels S., Koscielny S., Hill C. (2005). Prediction of cancer outcome with microarrays: A multiple random validation strategy. Lancet.

[81] Christensen E., Birkenkamp-Demtröder K., Sethi H., Shchegrova S., Salari R., Nordentoft I., Wu H.T., Knudsen M., Lamy P., Lindskrog S.V. (2019). Early Detection of Metastatic Relapse and Monitoring of Therapeutic Efficacy by Ultra-Deep Sequencing of Plasma Cell-Free DNA in Patients with Urothelial Bladder Carcinoma. J. Clin. Oncol..

[82] Powles T., Assaf Z.J., Davarpanah N., Banchereau R., Szabados B.E., Yuen K.C., Grivas P., Hussain M., Oudard S., Gschwend J.E. (2021). ctDNA guiding adjuvant immunotherapy in urothelial carcinoma. Nature.

[83] Powles T., Kann A.G., Castellano D., Gross-Goupil M., Nishiyama H., Bracarda S., Bjerggaard Jensen J., Makaroff L., Jiang S., Ku J.H. (2025). ctDNA-Guided Adjuvant Atezolizumab in Muscle-Invasive Bladder Cancer. N. Engl. J. Med..

[84] Paik S., Shak S., Tang G., Kim C., Baker J., Cronin M., Baehner F.L., Walker M.G., Watson D., Park T. (2004). A multigene assay to predict recurrence of tamoxifen-treated, node-negative breast cancer. N. Engl. J. Med..

