# Supplementary material for: A Programmed Cell Death Protein 1 Pathway-Derived Prognostic Transcriptomic Signature in Bladder Cancer: Development in TCGA-BLCA and Evaluation in Independent Cohorts
Source: Curr Issues Mol Biol. 2026 Oct 13;48(10):1056. doi: 10.3390/cimb48101056 (PMC13703632; doi:10.3390/cimb48101056)
Supplement: Supplementary file 1 [file cimb-48-01056-s001.zip › Supplementary_Materials_vB4_20261012.pdf]

A Programmed Cell Death Protein 1 Pathway-Derived Prognostic Transcriptomic Signature in Bladder Cancer: Development in TCGA-BLCA and Evaluation in Independent Cohorts

Supplementary Materials

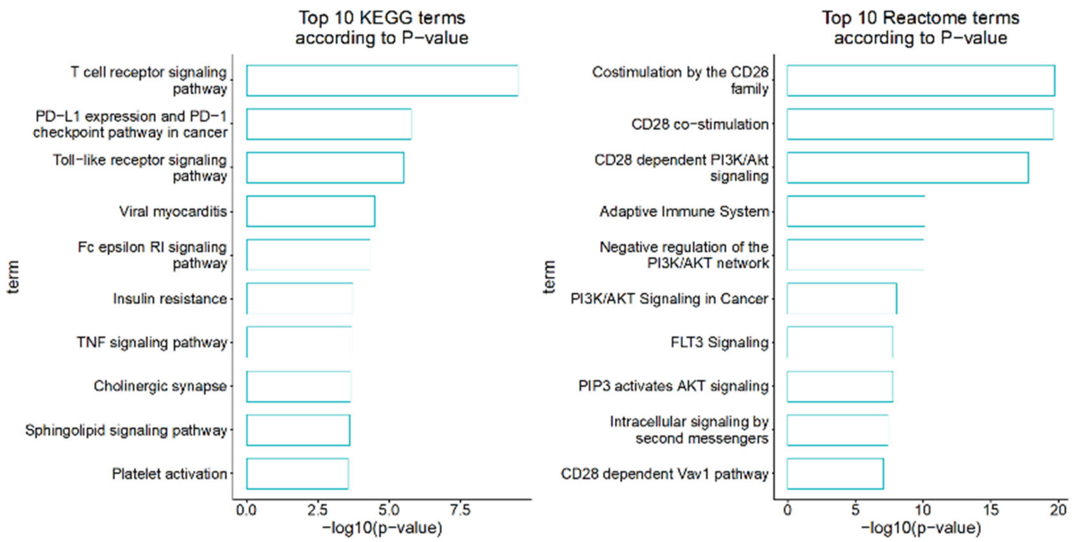

Figure S1. Functional annotation of the nine differentially expressed genes using the Reactome and the Kyoto Encyclopedia of Genes and Genomes (KEGG) databases.

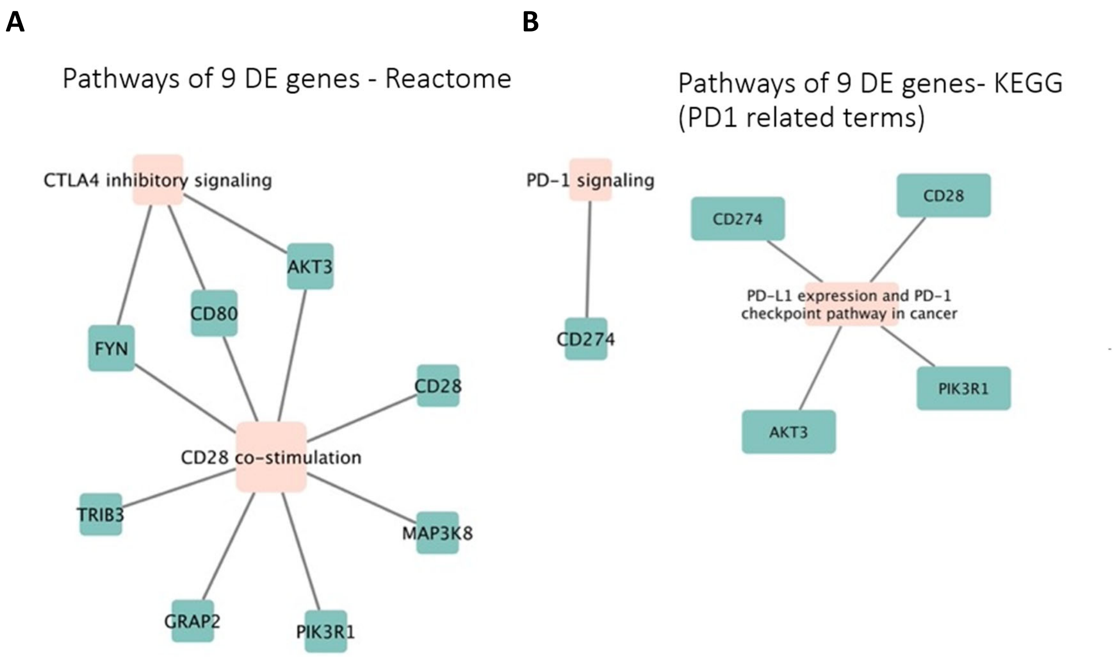

Figure S2. Pathway analysis of the nine differentially expressed genes: (A) top 10 Reactome terms and (B) KEGG terms ranked by p-value.

Table S1. Differential expression of the 68 PD-1 pathway genes between primary bladder cancer (n = 404) and normal bladder tissue (n = 19) in TCGA-BLCA, estimated with DESeq2 on raw read counts. Genes not quantified in the GDC STAR count matrix are shown as NA.

Entrez_id	Gene_symbol	log_2 fold change	*P* value	Adjusted *P* value
9020	*MAP3K14*	-0.8887	2.40E-05	1.53E-04
920	*CD4*	-0.5758	0.0710	0.1383
2534	*FYN*	-1.4975	1.33E-08	1.83E-07
5525	*PPP2R5A*	-0.9822	7.31E-15	3.26E-13
5526	*PPP2R5B*	-0.2828	0.0503	0.1049
998	*CDC42*	0.0556	0.5394	0.6529
5063	*PAK3*	-0.7101	0.1801	0.2885
5527	*PPP2R5C*	-0.6025	2.42E-09	3.89E-08
9402	*GRAP2*	-1.2311	4.90E-04	0.0021
57761	*TRIB3*	1.6772	2.09E-09	3.40E-08
1445	*CSK*	0.4293	6.28E-04	0.0026
5516	*PPP2CB*	-0.8721	2.23E-10	4.46E-09
208	*AKT2*	-0.0236	0.8315	0.8849
5518	*PPP2R1A*	0.1064	0.2931	0.4172
5296	*PIK3R2*	0.8882	0.0109	0.0295
1326	*MAP3K8*	-1.4034	1.90E-10	3.84E-09
5777	*PTPN6*	0.6680	4.44E-05	2.61E-04
5528	*PPP2R5D*	-0.0741	0.6565	0.7521
5515	*PPP2CA*	-0.2626	0.0068	0.0199
942	*CD86*	0.2326	0.4993	0.6180
10000	*AKT3*	-1.9588	4.51E-10	8.50E-09
8503	*PIK3R3*	-0.3989	0.0629	0.1255
79109	*MAPKAP1*	-0.4146	2.50E-04	0.0012
29126	*CD274*	1.1764	0.0058	0.0175
941	*CD80*	1.0874	0.0095	0.0264
5290	*PIK3CA*	-0.3164	0.0125	0.0332
5879	*RAC1*	0.2204	0.0565	0.1151
5519	*PPP2R1B*	0.4012	0.0025	0.0084
5170	*PDPK1*	-0.4137	0.0019	0.0067
7409	*VAV1*	-0.3896	0.2439	0.3637
207	*AKT1*	-0.0838	0.4451	0.5690
5295	*PIK3R1*	-1.1757	5.88E-07	5.55E-06
5058	*PAK1*	-0.1343	0.4506	0.5742

5529	*PPP2R5E*	-0.3942	1.33E-04	6.85E-04
117145	*THEM4*	0.1807	0.3919	0.5186
917	*CD3G*	-0.6674	0.0786	0.1503
1493	*CTLA4*	-0.2419	0.5562	0.6678
253260	*RICTOR*	-0.1211	0.4225	0.5481
915	*CD3D*	-0.7926	0.0515	0.1070
64223	*MLST8*	0.3459	0.0023	0.0079
7525	*YES1*	0.4454	0.0019	0.0066
2885	*GRB2*	0.0364	0.7087	0.7931
940	*CD28*	-1.1904	9.22E-04	0.0036
5781	*PTPN11*	-0.7475	2.88E-06	2.31E-05
3119	*HLA-DQB1*	0.3685	0.3725	0.4994
5062	*PAK2*	0.2113	0.0390	0.0852
3932	*LCK*	-0.5909	0.0860	0.1617
55615	*PRR5*	0.0182	0.9237	0.9504
5133	*PDCD1*	-0.3237	0.4733	0.5946
3123	*HLA-DRB1*	0.1995	0.6072	0.7119
3117	*HLA-DQA1*	0.3844	0.3647	0.4914
6714	*SRC*	0.7335	4.37E-04	0.0019
80380	*PDCD1LG2*	0.6355	0.1586	0.2616
3127	*HLA-DRB5*	0.1857	0.6568	0.7523
2475	*MTOR*	0.0204	0.8529	0.9006
919	*CD247*	-0.6729	0.0594	0.1198
916	*CD3E*	-0.9007	0.0234	0.0560
3122	*HLA-DRA*	0.1618	0.6690	0.7616
28682	*TRAV8-4*	-0.5487	0.2644	0.3863
28664	*TRAV19*	-0.6233	0.1426	0.2410
28653	*TRAV29DV5*	-0.1947	0.6931	0.7808
3115	*HLA-DPB1*	-0.2559	0.4857	0.6060
3113	*HLA-DPA1*	0.0013	0.9973	0.9982
3120	*HLA-DQB2*	0.1341	0.7426	0.8185
3118	*HLA-DQA2*	0.2267	0.6404	0.7385
4067	*LYN*	0.7065	0.0038	0.0121
28577	*TCRBV12S3*	NA	NA	NA
28589	*TRBV7-9*	-0.3109	0.5382	0.6517

Table S2. Discrimination, incremental value, calibration, and stability of the risk score in TCGA-BLCA.

Section	Measure	Value
Cohort	Patients with complete data	402
Cohort	OS events	176
Cohort	Median follow-up, months (reverse KM)	29.3
Cohort	Stage I/II vs III/IV	130 / 272
Continuous score	HR per SD, univariable	1.30 (1.17-1.45), p < 0.001
Continuous score	HR per SD, adjusted for age, sex, stage	1.24 (1.11-1.39), p < 0.001
Binary score (median)	HR high vs low, adjusted	1.75 (1.28-2.39), p < 0.001
Continuous score	Non-linearity test (restricted cubic spline, 4 knots)	chi2 = 6.58, df = 2, p = 0.037
Continuous score	Proportional hazards (Schoenfeld), risk score / global	p = 0.713 / 0.504
Harrell C-index (apparent)	Risk score alone	0.618 (0.575-0.662)
Harrell C-index (apparent)	Clinical (age, sex, stage)	0.630 (0.590-0.670)
Harrell C-index (apparent)	Clinical + risk score	0.664 (0.624-0.705)
Incremental value	Likelihood-ratio test, clinical vs clinical + score	chi2 = 12.13, df = 1, p < 0.001
Incremental value	Delta C-index (clinical + score minus clinical), 1000 bootstraps	0.035 (0.010-0.061)
Time-dependent AUC	Risk score alone 1-year	0.665 (0.602-0.728)
Time-dependent AUC	Risk score alone 3-year	0.615 (0.538-0.692)
Time-dependent AUC	Risk score alone 5-year	0.650 (0.560-0.740)
Time-dependent AUC	Clinical 1-year	0.659 (0.597-0.722)
Time-dependent AUC	Clinical 3-year	0.630 (0.559-0.702)
Time-dependent AUC	Clinical 5-year	0.634 (0.549-0.720)
Time-dependent AUC	Clinical + risk score 1-year	0.715 (0.654-0.776)
Time-dependent AUC	Clinical + risk score 3-year	0.668 (0.596-0.740)
Time-dependent AUC	Clinical + risk score 5-year	0.683 (0.600-0.765)
Internal validation (rms, clinical + score)	Optimism-corrected C-index	0.658 (apparent 0.664)
Internal validation (rms, clinical + score)	Calibration slope (optimism-corrected)	0.912
LASSO pipeline bootstrap	Bootstrap replicates	500
LASSO pipeline bootstrap	Replicates selecting exactly the published 7 genes	3.0%
LASSO pipeline bootstrap	Median number of genes selected	6
LASSO pipeline bootstrap	Apparent C-index of pipeline	0.607

LASSO pipeline bootstrap	Mean optimism	0.030
LASSO pipeline bootstrap	Optimism-corrected C-index of pipeline	0.577
Stage-stratified (adjusted for age, sex)	Stage I/II (n = 130, events = 35)	1.17 (0.81-1.69), p = 0.412
Stage-stratified (adjusted for age, sex)	Stage III/IV (n = 272, events = 141)	1.25 (1.11-1.40), p < 0.001
Stage-stratified (univariable, by AJCC stage)	Stage II (n = 128, events = 35)	1.26 (0.85-1.87), p = 0.255
Stage-stratified (univariable, by AJCC stage)	Stage III (n = 140, events = 57)	1.18 (1.01-1.38), p = 0.039
Stage-stratified (univariable, by AJCC stage)	Stage IV (n = 132, events = 84)	1.56 (1.23-1.96), p < 0.001
Stage-stratified (adjusted for age, sex)	Interaction risk score x stage group	p = 0.904

Expression data were re-derived from the Genomic Data Commons (STAR FPKM-UQ), and the published coefficients were applied unchanged. Models requiring stage used 402 patients with complete data (176 deaths). Clinical model: age (≥ 65 vs. < 65 years), sex, and stage (III/IV vs. I/II). Internal validation and calibration used 500 bootstrap resamples; the difference in C-index used 1000. The LASSO pipeline bootstrap repeated 10-fold cross-validated LASSO-Cox regression (lambda.min) on the nine candidate genes in each resample. HR, hazard ratio; SD, standard deviation; AUC, area under the time-dependent receiver operating characteristic curve.

Table S3. Bootstrap selection frequencies of the nine candidate genes (500 resamples of the LASSO pipeline).

Gene	Selection frequency	In published panel	Published coefficient
TRIB3	91.2%	Yes	1.298
MAP3K8	82.2%	Yes	-0.844
CD274	78.0%	Yes	-0.936
AKT3	76.6%	Yes	0.498
CD80	63.8%	Yes	-0.644
FYN	62.0%	Yes	0.957
PIK3R1	52.8%	No	-
GRAP2	52.2%	Yes	-0.134
CD28	33.2%	No	-

The exact published seven-gene set was selected in 3.0% of resamples; the median number of genes selected was six.

Table S4. Evaluation of the seven-gene risk score in TCGA-BLCA and all independent cohorts.

Cohort	Setting	Endpoint	n	Events	HR per SD (95% CI)	p	HR per SD, stage-adjusted	C-index (95% CI)	HR, training cutoff	Response AUC (95% CI)
TCGA-BLCA (training)	Mainly MIBC	OS	404	177	1.30 (1.17-1.45)	<0.001	1.24 (1.11-1.39)	0.618 (0.575-0.662)	1.97 (1.45-2.66)	
GSE13507	Mainly NMIBC	CSS (primary)	165	32	1.21 (0.83-1.77)	0.318	0.99 (0.73-1.35)	0.583 (0.478-0.689)	1.64 (0.79-3.41)	
GSE13507	Mainly NMIBC	OS	165	69	1.09 (0.83-1.43)	0.531	0.97 (0.76-1.24)	0.539 (0.463-0.615)	0.91 (0.57-1.46)	
GSE13507, log2-scale expression	Mainly NMIBC	CSS (sensitivity)	165	32	1.27 (0.87-1.85)	0.218	0.98 (0.71-1.36)	0.558 (0.454-0.662)		
GSE13507, stage I	NMIBC	CSS	103	3	1.22 (0.50-2.94)	0.664		0.652 (0.371-0.933)	1.96 (0.18-21.76)	
GSE13507, stage II-IV	MIBC	CSS	62	29	0.99 (0.65-1.52)	0.970		0.493 (0.388-0.597)	0.97 (0.45-2.10)	
GSE31684	MIBC, cystectomy	OS	93	65	0.87 (0.65-1.15)	0.320	0.80 (0.59-1.07)	0.515 (0.440-0.590)	0.92 (0.56-1.50)	
GSE48075	MIBC	OS	73	45	1.14 (0.82-1.60)	0.438	1.15 (0.81-1.64)	0.506 (0.395-0.617)	1.16 (0.64-2.08)	
GSE32894	Mainly NMIBC	DSS	224	25	1.24 (0.83-1.86)	0.300	1.19 (0.78-1.82)	0.530 (0.393-0.667)	1.41 (0.64-3.12)	
IMvigor210	Metastatic, atezolizumab	OS; response	348	232	0.91 (0.80-1.03)	0.149		0.502 (0.461-0.543)	0.96 (0.74-1.24)	0.474 (0.389-0.560)

GSE176307	Metastatic, PD-1/PD-L1 inhibitors	OS; response	88	57	1.37 (1.07-1.74)	0.011		0.583 (0.498-0.668)	2.00 (1.16-3.42)	0.678 (0.504-0.852)

The published coefficients and the standardization procedure were fixed before evaluation: expression values on each platform's linear scale were standardized per gene within each cohort and combined with the coefficients. The training cutoff is the TCGA-BLCA median of the risk score on the standardized scale, applied unchanged. Stage adjustment used stage III/IV vs. I/II, or stage II-IV vs. I in GSE13507 (with age and sex). Response AUC: area under the receiver operating characteristic curve for objective response, with the direction prespecified (higher score, lower response). CSS, cancer-specific survival; DSS, disease-specific survival; OS, overall survival; MIBC/NMIBC, muscle-invasive/non-muscle-invasive bladder cancer; SD, standard deviation.

Table S5. Immune-cell composition by risk group in TCGA-BLCA estimated with quanTIseq and MCP-counter.

**Method**	**Cell population**	**Median, high risk**	**Median, low risk**	**p**	**q**
quanTIseq	B cells	0.0053	0.0045	0.181	0.840
quanTIseq	Macrophages M1	0.0114	0.0121	0.877	0.877
quanTIseq	Macrophages M2	0.0293	0.0249	0.0264	0.264
quanTIseq	Monocytes	0.0000	0.0000	0.772	0.877
quanTIseq	Neutrophils	0.0352	0.0340	0.341	0.840
quanTIseq	NK cells	0.0124	0.0123	0.42	0.840
quanTIseq	T cells CD4	0.0000	0.0000	0.832	0.877
quanTIseq	T cells CD8	0.0020	0.0024	0.33	0.840
quanTIseq	Tregs	0.0131	0.0130	0.651	0.877
quanTIseq	Dendritic cells	0.0073	0.0091	0.782	0.877
MCP-counter	T cells	1.5925	1.7528	0.0138	0.046
MCP-counter	CD8 T cells	0.6716	0.8275	0.13	0.216
MCP-counter	Cytotoxic lymphocytes	0.8193	0.9410	0.157	0.225
MCP-counter	B lineage	2.0809	1.9391	0.493	0.548
MCP-counter	NK cells	0.2222	0.2580	0.0821	0.176
MCP-counter	Monocytic lineage	2.7314	2.5740	0.461	0.548
MCP-counter	Myeloid dendritic cells	1.7333	1.9529	0.0881	0.176
MCP-counter	Neutrophils	1.9631	1.9543	0.924	0.924
MCP-counter	Endothelial cells	2.5940	2.3510	0.000151	0.001
MCP-counter	Fibroblasts	6.5562	5.5454	8.11e-08	0.000

quanTIseq (TIL10 signature) estimates cell fractions from linear TPM values; MCP-counter estimates abundance scores from log2(TPM + 1) values. Groups were compared with the Wilcoxon rank-sum test; q-values are Benjamini-Hochberg-adjusted within each method. The original analysis used CIBERSORT, whose licensed files could not be obtained for the re-analysis.

Figure S3. Functional form of the association between the continuous risk score and overall survival in TCGA-BLCA.

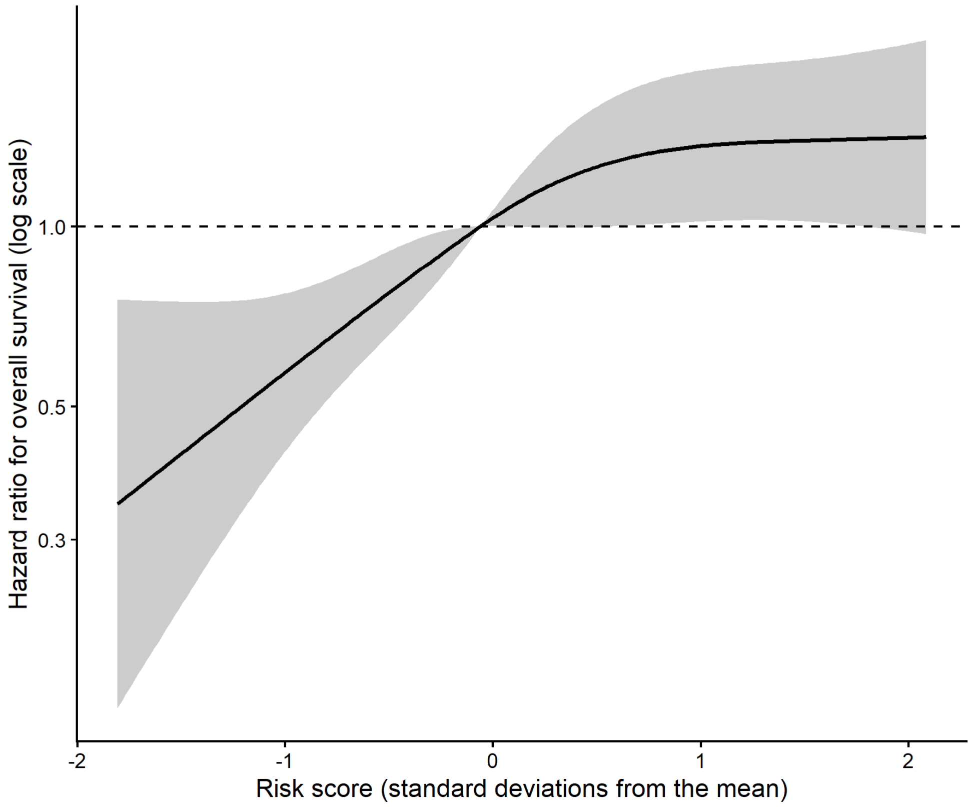

Restricted cubic spline with four knots, adjusted for age, sex, and stage (402 patients, 176 deaths). The solid line shows the hazard ratio relative to the mean risk score, and the shaded band the 95% confidence interval. Test for non-linearity p = 0.037.

Figure S4. Calibration of the clinical model plus risk score for 3-year overall survival in TCGA-BLCA.

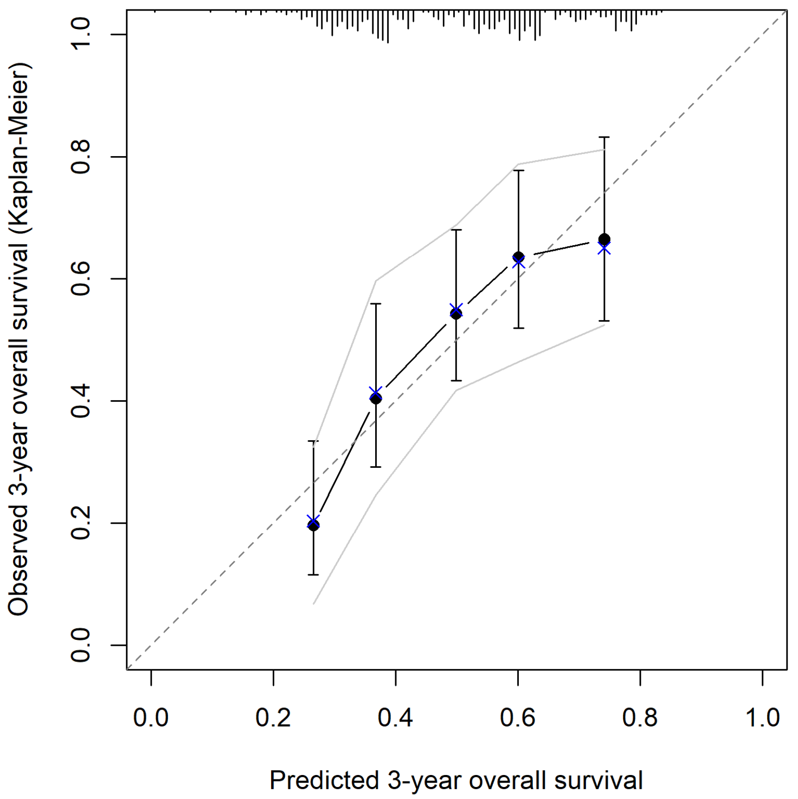

Points show the Kaplan-Meier observed 3-year survival against the predicted survival in groups of about 80 patients, with 95% confidence intervals; crosses show the bootstrap optimism-corrected estimates (500 resamples). The dashed line indicates perfect calibration.
